# Two Structural Motifs within Canonical EF-Hand Calcium-Binding Domains Identify Five Different Classes of Calcium Buffers and Sensors

**DOI:** 10.1371/journal.pone.0109287

**Published:** 2014-10-14

**Authors:** Konstantin Denessiouk, Sergei Permyakov, Alexander Denesyuk, Eugene Permyakov, Mark S. Johnson

**Affiliations:** 1 Biochemistry, Department of Biosciences, Åbo Akademi University, Turku, Finland; 2 Institute for Biological Instrumentation, Russian Academy of Sciences, Pushchino, Russia; University of Rome Tor Vergata, Italy

## Abstract

Proteins with EF-hand calcium-binding motifs are essential for many cellular processes, but are also associated with cancer, autism, cardiac arrhythmias, and Alzheimer's, skeletal muscle and neuronal diseases. Functionally, all EF-hand proteins are divided into two groups: (1) calcium sensors, which function to translate the signal to various responses; and (2) calcium buffers, which control the level of free Ca^2+^ ions in the cytoplasm. The borderline between the two groups is not clear, and many proteins cannot be described as definitive buffers or sensors. Here, we describe two highly-conserved structural motifs found in all known different families of the EF-hand proteins. The two motifs provide a supporting scaffold for the DxDxDG calcium binding loop and contribute to the hydrophobic core of the EF hand domain. The motifs allow more precise identification of calcium buffers and calcium sensors. Based on the characteristics of the two motifs, we could classify individual EF-hand domains into five groups: (1) Open static; (2) Closed static; (3) Local dynamic; (4) Dynamic; and (5) Local static EF-hand domains.

## Introduction

Calcium is essential for life [1] and plays at least two major roles in living organisms: structural and regulatory [2], [3]. Calcium is primarily found outside of cells, where it is complexed with phosphates or carbonates to form exo- and endoskeletons, serving as macro-scale structural scaffolds while also functioning to buffer the approximately 10^−3^ M extracellular levels of Ca^2+^ ions. In contrast, intracellular calcium concentrations are at least 10^4^ times lower, and require control mechanisms to be maintained at the appropriately low levels. Any failure of these control mechanisms may lead to sustained calcium overload and eventual cell and organ malfunction. The disparity in extracellular and cytoplasmic concentrations of Ca^2+^ ions supports the unique signaling and regulatory roles of calcium within the cell.

Calcium regulates many important aspects of cell activity, beginning with fertilization and ending with the apoptotic suicide of cells at the end of their life cycle [2]–[5]. Calcium ions are traditionally considered as secondary messengers liberated from intracellular and extracellular stores even though calcium itself may function to release Ca^2+^ ions from these stores. Calcium can also act as an extracellular primary messenger, thus taking on the role of a near-universal signaling molecule recognized by a wide variety of calcium-binding proteins in eukaryotes, prokaryotes and even viruses [2]–[6]. At the level of the protein structure, calcium may play important structural roles at the molecular level, required for maintaining appropriate conformations of individual proteins, such as at the β-propeller inter-domain interface in integrin α subunits and the homologous domains found in bacteria [7].

The scope of calcium ion functions extends to the regulation of contraction of all types of muscles, where binding of calcium to troponin C triggers the interaction of actin and myosin [8], [9]. As in most other cells, the concentrations of free Ca^2+^ ions in resting muscle is about 0.05–0.1 µM, but increases by one to two orders of magnitude when an external signal stimulates the cell. These Ca^2+^ ions are released either from intracellular stores (i.e. from the sarcoplasmic reticulum) or pumped up through sarcolemma from the intercellular space. In addition, increases in intracellular calcium concentrations play a central role in the function of neurons, triggering neurotransmitter release [10], [11]. Many aspects of neuronal activity, ranging from rapid modulation of channel function within a millisecond timescale to long-term switches in gene expression, are controlled by changes in the cytosolic calcium concentration. All of these various actions of Ca^2+^ ions are mediated by calcium-binding proteins that, in turn, interact with their target proteins. For example, regulation of transcription is coupled with numerous intracellular signaling processes often mediated by secondary messengers. Growing evidence points to the importance of calcium, as one of the most versatile second messengers, in activating or inhibiting gene transcription through actions frequently mediated by members of the EF-hand superfamily of calcium-binding proteins [12], [13].

Currently, there are eleven known calcium binding consensus motifs that proteins use to recognize Ca^2+^ ions, and whose sequence-variation profiles are established within the PROSITE database (http://prosite.expasy.org) [14]. The most commonly observed consensus motif for calcium binding is a characteristic DxDxDG calcium-binding loop [15], [16]. Among the 18 folds of proteins with the DxDxDG calcium-binding loop, there is a canonical EF-hand Ca^2+^-binding helix-loop-helix domain, where two antiparallel DxDxDG calcium-binding loops are flanked by interacting incoming and exiting α-helices [17]–[19]. The EF-hand domain is currently found in more than 71 non-redundant protein structures (see Homologous Superfamily EF-hand (1.10.238.10), the CATH database [20]) and is observed throughout all of the domains of life [17], [21]–[26]. We refer the reader to Gifford et al. (2007; Table 1 of the supplementary material in their article) for a listing of the range of functions performed by EF-hand containing proteins [17]. Given the wide range of functions of proteins with EF-hand calcium-binding domains, it is not surprising that these domains have been associated with numerous health issues, including Alzheimer's disease [27], cancer [28]–[30], neuronal disease [31], and disorders involving sodium ion channels in epilepsy, skeletal muscle disease, autism, and cardiac arrhythmias [32], [33].

**Table 1:** List of eleven non-redundant, representative calcium-bound X-ray and NMR protein complexes, which represent eleven different families of EF-hand domains.

N	Fold Family	Name of the Representative Protein, PDB Code, Resolution	Bound Cation	Refs.
1	Calbindin D9K	Calbindin D9K, PDB: 1IG5_A, R = 1.50 Å	One domain: 1 x Mg^2+^	[49]
2	S100 proteins	Calcyclin (S100), PDB: 1PSR_A, R = 1.05 Å	One domain: 1 x Ho^3+^	[50]
3	Polcalcin	Polcalcin phl p7, PDB: 1K9U_A, B, R = 1.75 Å	Two domains: 2 x Ca^2+^; 2 x Ca^2+^	[51]
4	Osteonectin	C-terminal (EC) domain of BM-40/SPARC/osteonectin, PDB: 1SRA_A, R = 2.00 Å	One domain: 2 x Ca^2+^	[52]
5	Parvalbumin	Parvalbumin, PDB: 2PVB_A, R = 0.91 Å	One domain: 2 x Ca^2+^	[53]
6	Calmodulin-like	Calmodulin, PDB: 1EXR_A, R = 1.00 Å	Two domains: 2 x Ca^2+^; 2 x Ca^2+^	[54]
7	Eps15 homology domain (EH domain)	Eps15, PDB: 1F8H_A, NMR Model 1	One domain: 1 x Ca^2+^	[55]
8	Cbp40 (plasmodial specific CaII-binding protein LAV1–2)	Cbp40 (plasmodial specific CaII-binding protein LAV1–2), PDB: 1IJ5_A, R = 3.00 Å	*apo*	[56]
9	Penta-EF-hand proteins	Grancalcin, PDB: 1K94_A, B, R = 1.70 Å	Three domains: 1 x Ca^2+^; 1 x Ca^2+^; *apo*	[57]
10	EF-hand modules in multidomain proteins	Cbl, PDB: 3BUX_B, R = 1.35 Å	*apo*	[58]
11	p25-alpha	Protein Cgi-38, PDB: 1WLM_A, NMR Model 1	*apo*	[59]

All of the structures share the same fold (Fold: EF Hand-like) and belong to the same EF-hand fold superfamily (Superfamily: EF-hand) (from SCOP [48]).

Conventionally, all EF-hand -containing proteins are divided into two groups: (1) Calcium sensors that include calmodulin, recoverin, and most of the other known EF-hand proteins, which function to translate the signal of a change in concentration of Ca^2+^ ions to various responses; and (2) Calcium buffers, represented by parvalbumin, calbindin D9k, calbindin D28k and calretinin that serve to modulate calcium signals as they bind free Ca^2+^ ions [17]. Calbindin-D28k and possibly calretinin, oncomodulin, and the mammalian β parvalbumin may have additional calcium sensor functions, leaving parvalbumin and calbindin-D9k as the only “pure” calcium buffers [34]. A typical hallmark for sensors is their relatively large calcium-dependent conformational changes, which are cooperative [35] and often accompanied by exposure of hydrophobic surfaces allowing the interaction with their target proteins [36]–[40].

During recent years, we have been examining the structural determinants that govern metal-controlled structural cooperativity, intrinsic disorder, dimer formation and various structural and chemical properties of parvalbumins and S100 proteins, which constitute the two largest sub-families of proteins with the Ca^2+^-binding EF-hand domain fold [41]–[44]. Here, we report two unique structural motifs found in all known families of the EF-hand proteins. These motifs provide a supporting scaffold for the calcium-binding DxDxDG sequence motif of the EF-hand. Each structural motif incorporates a cluster of three amino acids whose structure and structural rearrangement on calcium binding can serve to classify EF-hand domains into five separate classes.

## Results and Discussion

### Two Structural Motifs Outside of the Calcium Binding Regions Stabilize the EF-Hand Domain

**Figure 1:**
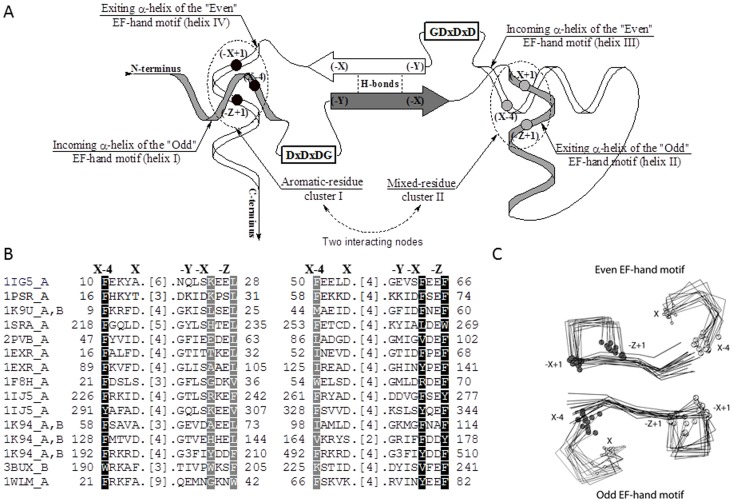
The structural elements of the EF-hand fold. Conserved elements of the EF-hand domains include (A) the flanking “incoming” and “exiting” α-helices (helix I to helix IV), the two interacting nodes, where the flanking α-helices interact and form interacting clusters I and II (shown in black and grey circles), position of the DxDxDG calcium binding loops, and the central β-sheet. (B) Structural alignment of the Odd and Even EF-hand motifs from the eleven fold representative structures. (C) Structural superposition of amino acids from Figure 1B. In 1K94_A, B, Figure 1B, “G3” designates two different three amino acid long insertions after Gly201 in 1K94_A and after Gly501 in 1K94_B. The structural frame of reference, “X”, “Y”, “Z”, “-X”, “-Y”, “-Z” and “X-4”, designate the seven key equivalent structural positions within all EF-hand domains, as numbered in Kretsinger and Nockolds [40]. The Odd EF-hand helix-loop-helix supersecondary structure is shown in grey. In panel A, B and C, the residues that belong to cluster I and cluster II are respectively highlighted in black and grey.

Nearly 40 years ago, Kretsinger and Nockolds (1973) described what came to be known as the canonical “EF-hand” Ca^2+^-binding supersecondary structure [45], the well-known helix-loop-helix structural unit that many proteins use for binding of calcium ions [17]–[19]. Since then, many different subfamilies of EF-hand proteins have been recognized [46] and the Protein Data Bank (PDB) currently lists more than 280 crystal structures and 130 NMR structures that contain one or several domains with the “EF hand-like” fold belonging to the “EF-hand” structural superfamily [47]. Table 1 lists representative crystal or NMR structures from the eleven different structural families of the EF-hand containing proteins (SCOP; Fold: EF hand-like; Superfamily: EF-hand) [48], where each domain consists of two antiparallel helix-loop-helix substructures. By convention, the substructures are named as the “Odd” (the substructure appearing first along the sequence; N-terminal) and “Even” (the substructure appearing second along the sequence; C-terminal) motifs and the N-terminal and C-terminal flanking α-helices in each helix-loop-helix substructure are respectively called the “incoming” and “exiting” α-helix (Figure 1A) [19], [46].

On the basis of their sequences, databases such as Pfam generally lump the Odd and Even motifs together as a single entity [60]. However, structurally, the motifs are different. While they have equivalent characteristic DxDxDG sequences within the Ca^2+^ ion binding loops and the β-strands forming the antiparallel β-sheet linker of the EF-hand domain (Figure 1A), their differences lie within the flanking α-helices.

Here, we have treated the Odd and Even EF-hand motifs as two separate structural entities, first examining 11 representative structures individually in order to identify equivalent structural positions shared within the Odd motif and within the Even motif (Figure 1B). Then, equivalent positions were used to superpose the EF-hand domains from the eleven different structural families listed in Table 1 (Figure 1C). The comparisons showed that the flanking α-helices are not only different for the Odd and Even EF-hand motifs, but that they also interact differently with each other, forming two different interacting nodes, each with a cluster of three residues, located at opposite ends of the central β-sheet linker (Figure 1A). The distinctive structural differences within the flanking α-helices of the Odd motif and of the Even motif contribute to the interactions that play a critical role in the formation, conformational dynamics and function of the EF-hand domain.

Kretsinger and Nockolds (1973) labeled the six residues that bind calcium in the carp muscle calcium-binding protein as X, Y, Z and -X, -Y, -Z [45], and we have used these positions as a convenient frame of reference for labeling other amino acid locations within the EF-hand motif. Because there are two helix-loop-helix EF-hand motifs – the Odd and Even motifs – interacting with each other in every EF-hand domain, each domain contains two sets of X, Y, Z and -X, -Y, -Z positions (Figure 1A).

When we examined the structural superposition of the eleven representatives of the EF-hand containing proteins we found conserved characteristic clusters of interacting residues located around positions X-4. Because the Odd and Even EF-hand motifs are antiparallel, there are two such clusters located on the opposite sides of the antiparallel β-sheet linker, which include interacting residues from equivalent positions X-4, -X+1 and -Z+1 (Figure 1A). Cluster I (solid black circles, Figure 1A) locks the ends of the EF-hand domain, i.e. the incoming α-helix of the Odd EF-hand motif (residue X-4) and the exiting α-helix of the Even EF-hand motif (residues -X+1 and -Z+1), while cluster II (solid grey circles, Figure 1A) locks the exiting α-helix of the Odd EF-hand motif (residues -X+1 and -Z+1) and the incoming α-helix of the Even EF-hand motif (residue X-4).

### Conservation of Amino Acids within the Two Clusters

The amino acids that constitute the two clusters are different. Cluster I is much less variable in sequence and mostly incorporates aromatic amino acids (phenylalanine, tryptophan, tyrosine): 15 out of 15 at X-4, 11/15 at -X+1 and 15/15 at -Z+1 (highlighted in black, Figure 1B). In contrast, cluster II includes a mix of aromatic, hydrophobic and polar amino acids of different sizes with 8/15 aromatic residues at X-4 (the rest are non-polar), 2/15 at -X+1 (others are variable in size and charge, e.g. glycine, lysine), and 4/15 at -Z+1 (the other residues are non-polar leucine and valine) (highlighted in grey, Figure 1B). In the one symmetric representative where one of three EF-hand domains is formed from homodimeric interactions (Ca^2+^-loaded human grancalcin; PDB code: 1K94), clusters I and II are obviously identical, fully aromatic, and no connecting loops are present (Figure 1B). The other two EF-hand domains in grancalcin are formed from a single-chain and are topologically asymmetric, similar to the other ten representative domains listed in Table 1.

Because of the differences in amino acid composition between the two clusters, we initially analyzed all interactions within them, with respect to whether they are considered stabilizing or destabilizing (Table S1 in File S1) according to the Automated Analysis of Interatomic Contacts software (LPC/CSU software) [61]. The LPC/CSU analysis reports that an equivalent number of non-polar stabilizing interactions could be seen in both clusters. However, cluster I, made up primarily of aromatic residues, almost completely lacked destabilizing interactions. In contrast, cluster II, composed of a wider mix of residue types, lacks some stabilizing interactions, made a larger number of destabilizing interactions, and even contained amino acids that did not directly interact with each other – i.e. an incomplete triad (Table S1 in File S1). For example, in the EF-hand of nucleoside diphosphate kinase (3BUX) tryptophan W202 (X-4) physically shields F205 from K225, preventing their interaction in cluster II. In osteonectin (1SRA), the interactions between H232 and F253 are absent as a result of a unique disulphide bridge, C256-C272, which joins the incoming and exiting α-helices of the even EF-hand helix-loop-helix motif. As a result of the disulphide bridge, the main-chain conformation and that of the F253 side chain are affected, resulting in a cavity filled with water (Wat429, Wat432 and Wat470 in 1SRA).

**Figure 2:**
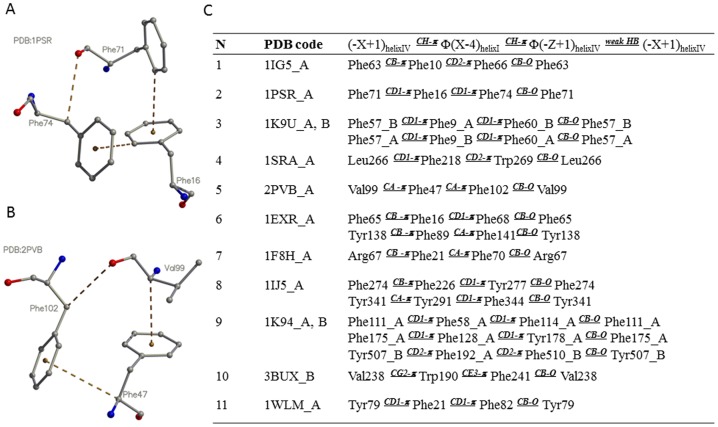
Example interactions within cluster I. (A) and (B) illustrate two types of interactions between the flanking α-helices I and IV (cluster I in Figure 1). The interactions occur *via* amino acids at positions -X+1, X-4 and -Z+1, which are also shown as black circles in Figures 1A and 1C and highlighted in black in the alignment in Figure 1B. Because in cluster I, positions X-4 and -Z+1 are purely aromatic (Φ) in all EF-hand representative structures, cluster I is called aromatic. (C) Contains the description of interactions for the eleven EF-hand fold representatives. The pattern “(-X+1)_helixIV_^CH-π^ Φ(X-4)_helixI_ ^CH-π^ Φ(-Z+1)_helixIV_ ^weak HB^ (-X+1)_helixIV_” indicates a circular interaction, where a side-chain atom of the –X+1 residue from the flanking α-helix IV forms a CH-π interaction with the ring of the X-4 aromatic amino acid from helix I, which, in turn, forms a CH-π interaction with the ring of the –Z+1 aromatic amino acid from helix IV, which interacts with the initial –X+1 residue from the flanking α-helix IV by means of a weak CH-O hydrogen bond.

Clusters I and II are thus different even though they are formed in both instances by the symmetrically similar positioned incoming and exiting α-helices. Unlike cluster II, cluster I has a predominant aromatic mini-core that is clearly stabilized by a set of linked CH-π and CH-O hydrogen bonds present in ten of the eleven representative structures (Figure 2; Tables S2 and S3 in File S1). The observed differences between the two clusters may reflect both structural and functional requirements: Reduced variability in cluster I may reflect a need for additional stabilization at the domains' open end – where the chains enter and exit the EF-hand – whereas cluster II may be inherently more stabile because a loop links and restrains the two ends of the EF-hand thus allowing for a more variable core. The difference also likely reflects functional and conformational requirements related to dynamic cooperative binding and release of Ca^2+^ ions and protein ligands.

In order to investigate the role of the two conserved but different clusters of amino acids stabilizing the structure of the EF-hand domain and the effect of calcium binding on the domain conformation, we have applied two different approaches: (1) For the eleven family representative structures, we calculated the total interacting surface areas among the amino acids within each cluster, as well as the interacting surface area between the two clusters within the domain (Table 2); (2) We also calculated the (i) interacting surface areas (Table 3; Table S4 in File S1) and (ii) the root mean-squared deviations of superpositioned clusters (RMSD; Table 4; Table S5 in File S1) for comparisons of all EF-hand domains found in the PDB (at the time when analyzed) whose structures are known for both the calcium-bound (*holo*; some also with bound target protein) and calcium-unbound (*apo*) forms. Individual values for all compared proteins are shown in Tables S4 and S5 in File S1.

**Table 2:** Summarized areas of interacting surfaces among the amino acids within clusters I and II (black and grey clusters in Figure 1, respectively), and the interacting area between the two clusters (I/II), within the EF-domains of the eleven representative structures.

Protein	Cluster I	Area, Å^2^	Cluster II	Area, Å^2^	I/II area, Å^2^
1IG5_A	F10, F63, F66	90.7	F50, K25, L28	74.9	18.1
1PSR_A	F16, F71, F74	97.0	F58, K28, L31	85.9	1.7
1K9U_A, B	F9_A, F57_B, F60_B	90.4	M44_B, L22_A, L25_A	45.7	NI
1K9U_A, B	F9_B, F57_A, F60_A	100.9	M44_A, L22_B, L25_B	53.6	NI
1SRA_A	F218, L266, W269	85.8	F253, H232, L235	50.4	40.8
2PVB_A	F47, V99, F102	81.5	L86, E60, L63	72.8	0.1
1EXR_A	F16, F65, F68	81.0	I52, T29, L32	51.1	NI
1EXR_A	F89, Y138, F141	75.1	I125, A102, L105	59.6	NI
1F8H_A	F21, R67, F70	56.9	W54, G33, V36	34.1	27.2
1IJ5_A	F226, F274, Y277	89.1	F261, R239, F242	104.1	0.4
1IJ5_A	Y291, Y341, F344	62.1	F328, K304, V307	67.7	28.7
1K94_A	F58, F111, F114	87.4	I98, A70, L73	43.3	34.2
1K94_A	F128, F175, Y178	85.2	V164, H141, L144	68.1	20.9
1K94_A, B	F192_A, Y507_B, F510_B	84.7	F492_B, Y207_A, F210_A	83.7	35.6
3BUX_B	W190, V238, F241	72.0	K225, W202, F205	83.1	45.8
1WLM_A	F21, Y79, F82	95.4	F66, G39, W42	81.0	43.6

NI, no interaction between the clusters.

**Table 3:** Effects of calcium binding on the conformation of all EF-hand domains whose structures are known in the *apo*-form and with bound Ca^2+^ ions, and target protein ligands.

	Cluster type	Proteins	Ligand	Area of Interactions, Å^2^	Comment
A	Cluster I (*not changed*)	All structures are shown in Table S4 in File S1	Apo-form	84±11	In all known structures, the conformation of aromatic cluster I does not change upon Ca^2+^ ion and ligand binding
	"	"	Bound Ca^2+^ ions	89±12	"
	"	"	Bound Ca^2+^ ions and bound target ligand	80±11	"
B	Cluster II (*not changed*)	Calbindin D9K, S100A16, S100P, Polcalcin, Parvalbumin, Oncomodulin, Calmodulin (N- and C-domains), Troponin C, Cbp40 (N- and C-domains), Calpain (N-domain)	Apo-form	68±12	Proteins where the conformation of cluster II does not change upon Ca^2+^ ion and ligand binding
	"	"	Bound Ca^2+^ ions	66±15	"
	"	"	Bound Ca^2+^ ions and bound target ligand	51±8	"
C	Cluster II (*changed*)	S100A1, S100A4, S100A5, S100A6, S100B, S100A13, Calpain (C-domain)	Apo-form	11±8	Proteins where the conformation of cluster II changes upon Ca^2+^ ion and ligand binding
	"	"	Bound Ca^2+^ ions	66±10	"
	"	"	Bound Ca^2+^ ions and bound target ligand	62±17	"
D	Cluster I/Cluster II (*rearranged*)	S100A4, S100A5, S100A6, S100B, Polcalcin, Calmodulin (N- and C-domains), Troponin C	Apo-form	33±14	Proteins where clusters I and II rearrange within the EF-hand domain upon Ca^2+^ ion and ligand binding
	"	"	Bound Ca^2+^ ions	3±3	"
	"	"	Bound Ca^2+^ ions and bound target ligand	8±11	"
E	Cluster I/Cluster II (*not rearranged*)	Calbindin D9K, S100P, Parvalbumin, Oncomodulin, Cbp40 (N-domain), Calpain (C-domain)	Apo-form	2±2	Proteins where clusters I and II do not rearrange within the EF-hand domain upon Ca^2+^ ion and ligand binding
	"	"	Bound Ca^2+^ ions	2±2	"
	"	"	Bound Ca^2+^ ions and bound target ligand	4±4	"
F	Cluster I/Cluster II (*not rearranged*)	S100A13, S100A16, Cbp40 (C-domain), Calpain (N-domain)	Apo-form	32±2	Proteins where clusters I and II do not rearrange within the EF-hand domain upon Ca^2+^ ion and ligand binding
	"	"	Bound Ca^2+^ ions	28±7	"
	"	"	Bound Ca^2+^ ions and bound target ligand	23±7	"

The interacting surface areas of amino acids within clusters I and II, and between the two clusters, are shown.

**Table 4:** Effects of calcium binding on the conformation of all EF-hand domains, whose structures are known in the *apo*-form and with bound Ca^2+^ ions, and target protein ligands (where they exist).

	Cluster type	Proteins	Ligand	RMSD Back- bone, Å	RMSD Heavy Atoms, Å	Comment
A	Cluster I (*not changed*)	All structures shown in Table S4 in File S1	(Apo-form)/(bound Ca^2+^ ions)	0.6±0.2	1.6±0.7	All known structures do not change conformation of Cluster I upon Ca^2+^ and target ligand binding
	"	"	(Apo-form)/(bound Ca^2+^ ions + target ligand)	0.8±0.3	2.0±0.9	"
B	Cluster II (*not changed*)	Calbindin D9K, S100A16, S100P, Parvalbumin, Calmodulin (N- and C-domains), Troponin C, Cbp40 (N- and C-domains), Calpain (N- and C-domains)	(Apo-form)/(bound Ca^2+^ ions)	0.6±0.3	1.3±0.5	Proteins that do not change conformation of Cluster II upon Ca^2+^ and target ligand binding
	"	"	(Apo-form)/(bound Ca^2+^ ions + target ligand)	0.7±0.3	1.2±0.5	"
C	Cluster II (*changed*)	S100A1, S100A4, S100A5, S100A6, S100B, S100A13, Polcalcin, Oncomodulin	(Apo-form)/(bound Ca^2+^ ions)	1.6±0.1	3.6±0.7	Proteins that change conformation of Cluster II upon Ca^2+^ and target ligand binding
	"	"	(Apo-form)/(bound Ca^2+^ ions + target ligand)	1.6±0.1	3.7±0.3	"
D	Cluster I/Cluster II (*rearranged*)	S100A16, Calmodulin (N- and C-domains), Troponin C	(Apo-form)/(bound Ca^2+^ ions)	1.7±0.2	2.3±0.5	Proteins that rearrange Clusters I and II within the EF-hand domain upon Ca^2+^ and target ligand binding
	"	"	(Apo-form)/(bound Ca^2+^ ions + target ligand)	1.5±0.3	2.4±0.9	"
E	Cluster I/Cluster II (*not rearranged*)	Calbindin D9K, S100P, Parvalbumin, Cbp40 (N- and C-domains), Calpain (N- and C-domains)	(Apo-form)/(bound Ca^2+^ ions)	0.8±0.3	1.3±0.5	Proteins that do not rearrange Clusters I and II within the EF-hand domain upon Ca^2+^ and target ligand binding
	"	"	(Apo-form)/(bound Ca^2+^ ions + target ligand)	0.8±0.3	1.2±0.4	"

The RMSD values between the *apo*-form and the protein with bound Ca^2+^ ions and the target ligand, are calculated using the back-bone atoms of the amino acids of the clusters, and separately, using all heavy atoms of the same amino acids. The RMSD data are shown for the superposition of clusters I and II separately (groups A–C), and for superposition of the two clusters, cluster I/cluster II, simultaneously (groups D and E).

*While groups A–C coincide between Tables 3 and 4, the RMSD calculations (this table) between clusters I and II were made only for the protein structures where the conformation of cluster II does not change upon calcium binding, and thus, proteins from group C were not included in D and E. This was done in order to observe only the inter-cluster conformational change.

### EF-Hand Family-Dependent Variation of the Two Conserved Clusters

The interacting surface area data from the fold representative structures (Table 2) show that the two clusters are distinctly different. Cluster I (with the exception of one domain in Cbp40 – plasmodial specific calcium-binding protein LAV1–2; 1IJ5) appears to be more compact, consistently having a larger interacting surface area than cluster II. In the representative fold structures, the interacting surface areas of cluster I are similar, regardless of whether the structure contains two (2PVB_A), one (1F8H_A) or no (1WLM_A) bound calcium atoms. In contrast, the interacting surface areas seen for cluster II vary significantly, from values closer to those of cluster I, reflecting a compact cluster, to values as low as 34.1 Å^2^.

The interacting area between clusters I and II varies from no contact in calmodulin to 34.2 Å^2^ in grancalcin and 45.8 Å^2^ in the EF-hand multi-domain protein Cbl. Where interactions occur between clusters I and II, the amino acid at positions -Z+1 from both clusters are involved and the exiting α-helices of the two EF-hand motifs are consequently positioned closer to each other.

### Effects of Calcium Binding on the Conformation of Individual Clusters Across Structures With and Without Bound Calcium

When considering all (19 in total) available pairs of EF-hand domains whose three-dimensional structures are known with and without bound calcium, the interacting surface area (Table 3) and RMSD (Table 4) values consistently show that interactions within cluster I are not altered dramatically on the binding of calcium or calcium plus a target protein (contact area with and without bound calcium averaging 89±12 Å^2^ and 84±11 Å^2^, respectively; RMSD for superpositioned Cα atoms of 0.6±0.2 Å; rows A in Tables 3, 4). Indeed, for both cluster I and cluster II the presence or absence of a protein ligand had little or no impact on the interactions within the cluster. In comparison to cluster I, the maximum contact surface area within cluster II averages about 20 Å^2^ less and in some cases the interacting surface area increases considerably on calcium binding. Cluster II can be divided into two groups. In one group, calcium binding produces little change in both the contact surface area within the cluster (68±12 Å^2^ → 66±15 Å^2^; row B in Table 3) and the local conformation (RMSD, 0.6±0.3 Å; row B in Table 4). In the other group, calcium binding leads to dramatically increased interactions among the three residues of cluster II (11±8 Å^2^ → 66±10 Å^2^; row C, Table 3) along with a notable conformational difference (RMSD, 1.6±0.1 Å; row C in Table 4).

### Effects of Calcium Binding on the Overall Fold of the EF-Hand Domain

The effects of binding of Ca^2+^ ions on the overall fold can be seen through the differences in the interacting surface areas between clusters I and II (column “B–G” in Table S4 in File S1), and RMSD values for the superpositioning of the two clusters as a unit (column “B–G” in Table S5 in File S1). Both sets of comparisons indicate that all EF-hand domains are divided into either those that do not change the relative position between clusters I and II: 2±2 Å^2^ (apo) → 2±2 Å^2^ (Ca^2+^) (row E in Table 3), and 32±2 Å^2^ (apo) → 28±7 Å^2^ (Ca^2+^) (row F in Table 3), and with an RMSD of 0.8±0.3 Å (row E in Table 4); or those that change their conformation upon calcium binding, 33±14 Å^2^ (apo) → 3±3 Å^2^ (Ca^2+^) (row D in Table 3), and the RMSD of 1.7±0.2 Å (row D in Table 4).

### Effects of Calcium Binding on the Conformation of Individual Clusters and the Overall Fold of the EF-Hand Domains, which bind single Ca^2+^ ion

All 19 pairs of EF-hand domains, whose conformational changes upon calcium binding are analyzed and shown above in Tables 3 and 4, require two Ca^2+^ ions in two defined calcium binding sites. However, there is also a small group of domains with the single calcium binding EF-hand motifs. Currently, there are only four such proteins, whose three-dimensional structures are known with and without bound calcium. These four proteins incorporate six different EF-hand domains shown in Table 5.

**Table 5:** Single calcium binding EF-hand domains with known *apo*- and *holo*-form structures.

PDB code	Ligand	Target	Cluster I	Surf.	Cluster II	Surf.	I/II surf.	Refs.
Grancalcin, EF1-EF2 domain								
1K95_A	-	-	F58, F111, F114	96.9	I98, A70, L73	46.7	22.3	[64]
1K94_A	1 Ca^2+^	-	F58, F111, F114	87.4	I98, A70, L73	43.3	34.2	[64]
Grancalcin, EF3-EF4 domain								
1K95_A	-	-	F128, F175, Y178	91.0	V164, H141, L144	71.9	28.5	[64]
1K94_A	1 Ca^2+^	-	F128, F175, Y178	85.2	V164, H141, L144	68.1	20.9	[64]
Myosin essential light chain								
3JTD_C	-	-	F15, F65, F68	108.1	G54, A30, L33	50.3	32.2	[65]
3JVT_C	1 Ca^2+^	-	F15, F65, F68	93.0	G54, A30, L33	41.9	43.9	[65]
Recoverin, N-domain								
1IKU_A	-	-	F35, F83, Y86	41.8	F70, R46, F49	68.9	63.6	[66]
1JSA_A	1 Ca^2+^	-	F34, F82, Y85	58.8	F69, R45, F48	79.3	22.0	[67]
Recoverin, C-domain								
1IKU_A	-	-	F106, E169, F172	82.0	W156, K119, V122	120.9	6.4	[66]
1JSA_A	1 Ca^2+^	-	F105, E168, F171	94.2	W155, K118, V121	63.7	NI	[67]
Troponin C, cardiac								
1SPY_A	-	-	F24, F74, F77	71.5	I61, T38, L41	67.0	38.5	[68]
1AP4_A	1 Ca^2+^	-	F24, F74, F77	70.7	I61, T38, L41	84.0	8.0	[68]
2MKP_C	1 Ca^2+^	Troponin I	F24, F74, F77	69.1	I61, T38, L41	18.7	20.2	[69]
2KRD_C	1 Ca^2+^	Troponin I, WW7	F24, F74, F77	76.4	I61, T38, L41	78.8	NI	[70]
1LXF_C	1 Ca^2+^	Troponin I, BEP	F24, F74, F77	119.5	I61, T38, L41	18.9	NI	[71]

Summarized areas of interacting surfaces among the amino acids within cluster I and cluster II (Figure 1), and the interacting area between the two clusters (I/II) are shown. NI, no interaction between the clusters.

Similarly to the EF-hand domains with two Ca^2+^ ions, the single calcium binding EF-hand motifs show that interactions within cluster I are not altered dramatically on the binding of calcium or calcium plus a target protein (row A in Table 3; and Table 5). Moreover, with the exception of recoverin (C-domain), also the conformational changes within the cluster II and the relative conformation between clusters I and II do fall within the categories of double calcium binding EF-hand motifs (Table 3). For example, in all known EF-hand domains of grancalcin and the myosin essential light chain, the conformation of cluster II and the relative conformation between clusters I and II do not significantly change upon calcium binding, as seen, for example, in the calbindin D9K calcium buffer (rows B and E in Table 3; and Table 5). On the other hand, recoverin (N-domain) and troponin C (cardiac) do rearrange the relative conformation between clusters I and II and undergo domain opening, similar to the N- and C-domains of calmodulin (rows B and D in Table 3; and Table 5).

A notable exception from the behavior of double calcium binding EF-hand motifs is shown by the recoverin, C-domain. Calcium binding to this protein results in significant weakening of interactions within the cluster II as opposed to all the other known single or double calcium binding EF-hand motifs descried above, where cluster II becomes tighter and starts resembling cluster I. The domain conformation of recoverin (C-domain) is already open without bound Ca^2+^ ion, and remains open after the Ca^2+^ ion is bound.

### Clusters I and II and a Short β-Sheet Linker Form the Hydrophobic Core of the EF-Hand Domain

**Figure 3:**
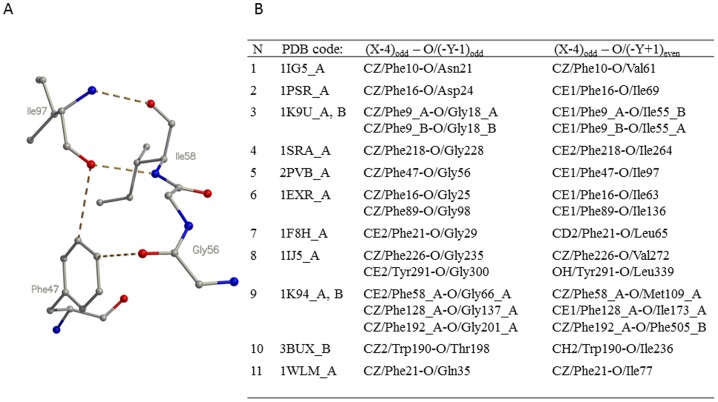
Interaction of cluster I with the central β sheet. The side chain of the aromatic residue at position X-4 in cluster I is perpendicular and directly interacts with the β-sheet linker of the EF-hand domain, contributing to its hydrophobic core. (A) Interactions between residue X-4 of cluster I (F47) and the β-sheet linker in 2PVB. (B) The interactions in the fold-representative structures include two classic, weak CH-O hydrogen bonds, whose detailed parameters are given in Table S6 in File S1. The (X-4)_odd_ – O/(-Y-1)_odd_ interaction shows the weak CH-O hydrogen bond between residue X-4 of cluster I and residue -Y-1 (G56 in 2PVB) of the “odd” EF-hand motif, while the (X-4)_odd_ – O/(-Y+1)_even_ interaction shows the weak CH-O hydrogen bond between the same residue X-4 of cluster I and residue -Y-1 (I97 in 2PVB) of the “even” EF-hand motif.

Clusters I and II join the incoming and exiting helices of the EF-hand domain structure and predominantly consist of non-polar and hydrophobic amino acids. The short β-sheet linker of the EF-hand domain is also mainly hydrophobic and is formed from two β-strands bound together by two hydrogen bonds (shown as dashed lines, “H-bonds” in Figure 1A). Both clusters interact with the β-sheet linker and together contribute to the hydrophobic core of the EF-hand. The side chain of the central amino acid (X-4) of cluster I (e.g. F47 in 2PVB) in all eleven representative structures of the EF-hand domain is always positioned in close proximity and perpendicular to the plane of the β-sheet linker (I58 and I97 in 2PVB) (Figure 3). The side-chain ring of residue X-4 forms two CH-O close contacts with the β-strand and the contact distance and angle parameters of the contacts satisfy criteria for classic weak CH-O hydrogen bonds (Table S6 in File S1) [63].

Because the amino acid at X-4 in cluster II is not strictly aromatic, but sometimes hydrophobic or even polar (Figure 1B), several types of interactions take place with the β sheet. If the central amino acid X-4 is an aromatic residue, interactions with the short β-sheet linker are similar to those for cluster I, and both clusters contribute to the EF-hand hydrophobic core. Where residue X-4 is not aromatic, the interactions with the β-sheet linker vary from being polar (e.g. multi-domain protein Cbl, 3BUX_B, where the side-chain NZ atom of K225 interacts with the side-chain OD1 atom of D234 through the HOH445 water molecule) to being weak Van der Waals interactions (e.g. parvalbumin, 2PVB, where the L86 side chain interacts with the Cα atom of G95 at 4.5 Å). In the representative structures the side chain of residue X-4 of cluster II points directly towards and interacts with the β-sheet linker, contributing to hydrophobic core's stability (Figure 3).

### Biological Implications

#### EF-Hand Domain Clusters I and II are Structurally and Functionally Non-Equivalent

Regardless of the outward symmetry in the folding pattern of the EF-hand domains (Figure 1A), the two halves of the domain have been shown experimentally to be non-equivalent and non-interchangeable, and thus asymmetrical. Lakowski et al. (2007) engineered and refolded calmodulin creating a “reversed” calmodulin fragment starting with the even helix-loop-helix EF-hand motif from the N-terminal EF-hand domain, including the inter-domain region, and ending with the odd helix-loop-helix EF-hand motif from the C-terminal EF-hand domain [72]. As a result, this EF-hand construct would have the fold of the EF-hand domain, as in calmodulin, with similar flanking α-helices and the short β-strand linker, but the locations of clusters I and II would be swapped. These structural changes led to ∼100 times weaker binding of the first calcium atom and 3000 times weaker binding of the second calcium atom, rendering the calmodulin fragment inactive [72]. It may be a general requirement of EF-hands that cluster I must have a more static structure with a high level of mutual interactions because it is located where the polypeptide chains enter and leave the domain. In contrast, cluster II has a constraining 5–15 residue loop [19] joining the two helices where residues of cluster II are located and may account for the higher degree of amino acid variability in comparison with cluster I. Variability at cluster II may also play an important role and be necessary in order to allow for different degrees of conformational change on calcium binding and release that are directly associated with the molecular and biological function.

#### Clusters I and II, and Nearby Residues are Critical for Both Structure and Function

The functional importance of the region adjacent to the calcium-binding motif can also be seen through the effects of naturally-occurring and engineered mutations. There are a number of EF-hand containing proteins that are linked with health issues [27], but only a few mutations have been clearly established as being directly linked to a particular condition. For example, in guanylate cyclase activating protein 1 (GCAP1) [73] polymorphisms have been directly associated with autosomal dominant cone dystrophy: i.e. Y99C at position X-1 of the odd EF-hand motif [74] and the replacement of asparagine-threonine for I143 (X-1) of the even EF-hand motif [75].

Engineered mutations have also pointed to the functional importance of clusters I and II and nearby residues. Koltzscher and Gerke (2000) have shown that for the calcium buffer S100P an F15A mutation at X-4 in cluster I completely abolished dimerization required for activation [76]. In Calbindin D9K (fold family 1 in Table 1; 1IG5_A in Figure 1B), the mutation of amino acids to alanine at X-4 (F10) and -Z+1 (F66) in cluster I and -Z+1 (L28) in cluster II significantly reduced functional calcium binding whereas the mutation of phenylalanine at -Z+1 to the larger aromatic tryptophan increased calcium binding 25-fold (Kragelund et al., 1998) [77]. Tikunova et al. (2002) examined the role of 27 hydrophobic residues within the EF-hand domain by replacing these residues with polar glutamine in the F29W activating mutant of the N-domain of troponin C (N-TnC, calmodulin-like family, fold family 6 in Table 1) [78]. The largest observed effects were the 123-fold decrease in the K_d_ for F26QTnC^F29W^ at X-4 in cluster I, followed by I37QTnC^F29W^ within the β-strand linker (↓24%), I62QTnC^F29W^ at X-4 in cluster II (↓12%), and F78QTnC^F29W at^ -Z+1 in cluster I (↓8%), and similar 6 to 7 -fold changes at I73, V80 and M81, but small effects on -X+1 (F75) in cluster I and -Z+1 (L42) in cluster II. Increases in the K_d_ were seen for V45QTnC^F29W^ (↑19%) and L49QTnC^F29W^ (↑19%) also located the exiting helix of the odd EF hand motif. The packing of the hydrophobic triad in the vicinity of cluster I of the C-terminal calmodulin-like domain (fold family 6, Table 1; PDB code 4DCK) was shown to be critical for the normal functioning of the sodium channel of Na_V_1.5 [33]. The degree of likely disruption to the local hydrophobic environment was associated with the severity of effects on channel gating by mutations of Y1795 at X-7 (incoming helix of the odd motif), for which inherited mutations cause congenital cardiac arrhythmias, and X-4 (W1798) and -Z+1 (I1853) within cluster I. In the ALG-2 protein (PDB code: 2ZN9; penta-EF-hand protein family; family fold 9, Table 1), the F148S mutation at cluster I alters the wild-type binding site recognition for the protein ligand phospholipid scramblase protein PLSCR3 [79].

#### Clusters I and II Can Serve as Markers for EF-Hand Domain Types

EF-hand domains have been classified as buffers and as sensors [36], [80], [81]. Sensors such as calmodulin and troponin C [34] as well as S100A1, S100A4, S100A6 and S100B [82] would involve communication with a protein ligand and involve a calcium-sensitive conformational change in order to expose hydrophobic residues and thus mediate the interaction. In contrast, buffers (signal modulators) [81] would not, and a lack of a conformational change within the EF-hand domain would indicate that a domain was a buffer. For example, S100A16, whose structure did not show any significant conformational change upon calcium binding [83]. Similarly, no significant structural changes on calcium binding were observed for the calpain C-terminal domain [84], Cbp40 [85] and S100P [86] and they would be considered as likely buffers on this basis.

The structural changes refer to the transition between “closed” and “open” protein conformation, which may or may not occur on calcium binding, and the resulting calcium-dependent/independent or calcium-sensitive/insensitive mechanism of protein function. Originally, it was shown that in calmodulin and troponin C, distances and angles between the flanking α-helices did change upon calcium binding, while in calbindin 9K they did not [19]. This calcium dependent or independent behavior of EF-hand domains was later extended to other EF-hand proteins, where the domains were examined to see whether they would undergo structural rearrangements similar to those seen in calmodulin and troponin C (calcium-sensitive or sensor mechanism) or would show no structural changes as seen in calbindin 9K (calcium-insensitive or buffer mechanism) [19], [82]. However, as Schwaller (2009) notes, “pure” calcium buffers like calbindin D9K and parvalbumin are few, as the buffers calbindin-28K, oncomodulin and calrectinin may also have calcium sensor functions [34].

**Figure 4:**
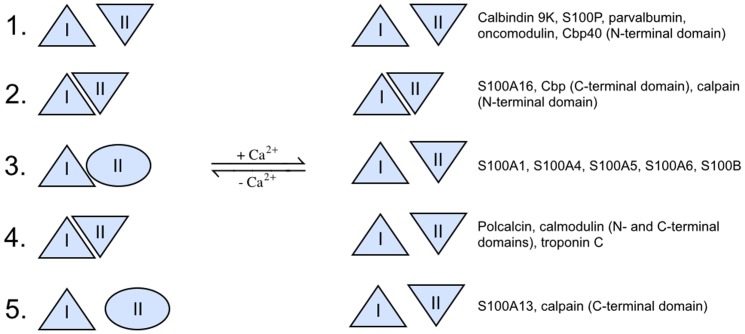
Five distinguishable groups of EF-hand domains based on the degree of structural rearrangements within clusters I and II, and the inter-cluster interactions that take place upon calcium binding: (1) Open Static (open domain conformation and no conformational changes); (2) Closed Static (closed domain conformation and no conformational changes); (3) Local Dynamic (simultaneous conformational changes in clusters I and II and the entire domain); (4) Dynamic (only global conformational changes, but not in clusters I and II); and (5) Local Static (stable open domain conformation, conformational changes only in clusters I and II, but not the entire domain). Domain level conformational changes, are shown by the small (“closed” domains) and large (“open” domains) distance between clusters I and II (triangles, ellipses and inverted triangles). Domain types (3) and (4) do undergo domain opening, while domain types (1), (2) and (5) do not. The conformation of the domains of type (2) remains “closed”, while the conformation the domains of types (1) and (5) remains open. Local conformational changes in clusters I and II are shown by normal triangles (compact conformation of cluster I), inverted triangles (compact conformation of cluster II), and ellipses (less compact, more open conformation of cluster II). The conformation of cluster I does not change in all of the known structures and is the same in buffers and sensors, such as in calbindin 9K, calmodulin and troponin C. The conformation of cluster II does change from being less compact to more compact in the domains of types (3) and (5). In group (4), EF-hand domains undergo domain opening, but the conformations of the conserved clusters I and II remain intact.

The differences observed among pairs of structures with and without bound calcium have allowed us to effectively separate and classify EF-hand domains into five separate groups (Figure 4). The quasi-symmetry of the EF-hand domain belies the fact that the component parts, with the rare exception of homodimeric interactions, are not identical and not truly symmetrical and that the domain seen across different proteins can have a largely static structure or one that rearranges considerably on binding Ca^2+^ ions. As a key underlying component of the EF-hand domain structure, the three-residue clusters, I and II, are critical for the structure of the EF-hand domains they are found within and consequently the overall function of the protein. Cluster I is primarily aromatic and as a unit it appears to be the more static and stabile linchpin for the overall structure since it maintains higher mutual surface area contacts that do not rearrange dramatically on calcium binding in any of the available structures, varying between 80±11 Å^2^ and 89±12 Å^2^ (row A, Table 3). In contrast, cluster II is more variable in sequence, the interaction surface area is lower than for cluster I, ranging between 11±8 Å^2^ (row C, Table 3) and 68±12 Å^2^ (row B, Table 3), and the contacts can reorganize significantly when Ca^2+^ ions are bound or released. Thus, it would appear that the makeup of cluster II is important for the functional requirements of the individual EF-hand.

The interaction surface area for cluster II would correlate with the function of the EF-hand according to the buffer-sensor classification as follows. If the interaction area within cluster II, *apo* form, is small (row C, Table 3), then this points towards the domain being a calcium sensor. If the interaction surface area between clusters I and II for the *holo* form is small (rows E and F, Table 3), then it is likely that the domain is a calcium buffer.

#### Individual EF-Hand Domains can be Classified into Five Groups Based on the Characteristics of Clusters I and II

Altogether, the EF-hand domains fall into five distinguishable groups based on the degree of structural rearrangements that take place upon calcium binding (Figure 4). In all five types the three residues of cluster I form mutually compact interactions.

Type 1. Open static EF-hand domains – where the domain conformation is open, cluster II has compact interactions, and clusters I and II do not interact. These features are not affected by the presence or absence of calcium. Observed for the structures of EF-hand domains considered to function as buffers: calbindin 9K, S100P, parvalbumin, oncomodulin and Cbp40 (N-terminal domain).

Type 2. Closed static EF-hand domains – where the domain conformation is closed, cluster II is compact, and clusters I and II are close to each other and interact with each other. These features are not affected by the presence or absence of calcium. These features are shared by the calcium-free structures of the Type 4 set (see below). Includes the buffers S100A16, Cbp40 (C-terminal domain) and calpain (N-terminal domain).

Type 3. Local dynamic EF-hand domains – where a closed-to-open transition takes place on calcium binding in which the cluster II becomes more compact and interactions between clusters I and II are eliminated. Includes the calcium sensors, such as S100A1, S100A4-A6 and S100B.

Type 4. Dynamic EF-hand domains – where cluster II is compact in the absence of calcium but where the inter-cluster distance increases on calcium binding. Includes the classical calcium sensors such as calmodulin (N- and C- domains) and troponin C, and polcalcin.

Type 5. Local static EF-hand domains – where the conformation of cluster II becomes more compact on calcium binding but the inter-cluster distance remains essentially unaltered. Includes the calcium sensors S100A13 and calpain (C-domain).

With the exception of the Type 2 domains, all other domains on calcium binding have clusters I and II that are compact and are separated from each other (Figure 4). The clearly different functional role of clusters I and II in calcium buffers and sensors explains why the engineered swap of the clusters in the EF-hand domain construct of calmodulin led to the loss of function described by Lakowski et al. [72]. As a consequence of these comparisons of EF-hand domain structures, it should be possible to classify some EF-hands based on a single example of a three-dimensional structure. For structures with bound calcium, closed static EF-hand domains of Type 2 can be differentiated from the other four types; whereas, in the unbound state it would be possible to identify domains belonging to Types 1, 3 and 5. The overall conformation of EF-hand domains are highly variable [37], [81]; but, when considered in terms of the two local, three-residue clusters that help maintain the domain structure, it is possible to classify individual proteins into discrete groupings. Detailed summary on the datasets after classifying them into five groups, including characteristic features of the classes, as well as PDB and sequence IDs (http://www.uniprot.org) is shown in Table S7 in File S1.

## Materials and Methods

Currently, there are eleven known calcium binding consensus motifs [14], whose sequence-variation profiles are established within the PROSITE database [87]. The EF-hand motif is currently found in more than 71 non-redundant representative domains (see Homologous Superfamily EF-hand (1.10.238.10), the CATH database [20]). We have extracted and analyzed more than 280 (all) crystal structures and 130 NMR structures currently listed in the Protein Data Bank (PDB) [47] that contain one or several domains with the “EF hand-like” fold belonging to the “EF-hand” structural superfamily, according to the SCOP database (SCOP; Fold: EF hand-like; Superfamily: EF-hand) [48].

From 410 available X-ray and NMR structures of EF-hand domains, we had to create three different datasets: (1) a representative non-redundant dataset of different EF-hand domains to scan for structural motifs, i.e. clusters of amino acids and interactions, which are conserved across different structural families of EF-hand proteins; (2) a dataset to study effects of calcium binding on the domain conformation of double calcium binding EF-hand motifs, which would include all known pairs of EF-hand domains, whose three-dimensional structures are known with and without two bound calcium atoms; and (3) a similar dataset to study calcium binding effects on single calcium binding EF-hand motifs. For the first task of searching for conserved amino acid clusters and interactions, we had to manually choose a set of best resolution representative structures from the 11 different structural families of EF-hand proteins, given in SCOP [48]. Where a family contained only NMR structures, a most represented and complete NMR structure was chosen (fold families 7 and 11, Table 1). The search for structural motifs and creation of the first dataset involved a semi-manual pairwise and multiple global and local structural comparisons of the 11 representative structures using the Accelrys Discovery Studio molecular modeling environment (www.accelrys.com). Because of semi-manual all-against-all structural comparisons and analysis of local structural similarities, we stayed at the structural family level (11 structures), and did not go below, to the level of individual protein domains (51 structures). Beyond the 51 structures of individual protein domains, the rest of the 410 structures included either orthologs of the same proteins from the different species or structures of various mutants, which could be discarded from analysis. For the second and third tasks to study effects of calcium binding on the domain conformation, the creation of two different datasets was straightforward. We manually analyzed all 410 structures of EF-hand domains and the corresponding literature to select all double and single calcium binding EF-hand domains, whose three-dimensional structures are known with and without bound calcium and possibly other ligands.

All types of structural superpositions and RMSD calculations based on back-bone and all atoms other than hydrogen, including (1) the superposition of the entire Odd and Even EF-hand motifs of EF-hand containing proteins; and (2) local structural superpositions of clusters I and II, were done using the SuperPose superposition server [88] and the Accelrys Discovery Studio molecular modeling environment (www.accelrys.com).

Calculation of atomic contacts and interacting surface areas was done using the Contacts of Structural Units (CSU) software, which is based on the surface complementarity approach developed by Sobolev et al. [61]. The “Detailed Analysis” procedure within the CSU software was used to calculate bond and surface parameters for all amino acids of the clusters individually.

Geometric parameters to assign CH-π interactions were chosen to satisfy criteria given in Brandl et al. [62]. If X designates the center of an aromatic ring, then the C-X distance must be ≤4.5 Å; the C-H-X angle must be greater than 120°; and the d_Hp-X_ projection distance must be ≤1.2 Å [62]. We used an additional criteria, d(H-X) ≤3.5 Å, to ensure that the CH group points directly to the center of the π-ring (Table S2 in File S1).

The criteria to assign weak hydrogen bonds were taken from Derewenda et al. [63]. Firstly, the C-H-O angle ζ must be greater than 90°. Secondly, an electronegative atom must be located adjacent to the carbon atom, such that the acidity of hydrogen atoms attached to the carbon atom increases, and consequently, the carbon atom could be a hydrogen bond donor. Thirdly, the C-O distance must be ≤4.0 Å and the H-O distance must be ≤3.0 Å. Two distance criteria d(N-O) ≤3.7 Å and d(H-O) ≤2.7 Å were used for the conventional hydrogen NH-O bonds; angular criteria, as described above, were also imposed.

All geometrical calculations (i.e., angles, torsion angles and distances) were made using the Accelrys Discovery Studio molecular modeling environment. Color figures in this manuscript were produced with MOLSCRIPT [89] and Raster3D [90].

## Supporting Information

File S1 Contains Tables S1–S7. **Table S1**. Assignment of interatomic contacts among the residues in clusters I and II according to the Interatomic Contacts software [61]. The “+” and “−” signs show presence or absence of Stabilizing and Destabilizing contacts, respectively, as defined by the Interatomic Contacts program. NI, absence of any interactions between the amino acids. 1EXR and 1IJ5 contain two EF-hand domains within chain A, while 1K94 contains two EF-hand domains within chain A, and one additional domain, shared between chains A and B. **Table S2**. Values of distances and angles for the CH-π interactions shown in Figure 2 for the interactions within cluster I. All designations are as in [62]. **Table S3**. Values of distances and angles for the CH-O interactions shown in Figure 2 for the interactions within cluster I. All designations are as in [63]. **Table S4**. Summarized areas of interacting surfaces among the amino acids within cluster I and cluster II (Figure 1), and the interacting area between the two clusters (I/II), for all EF-hand domains with known apo- and holo-form structures. NI, no interaction between the clusters. **Table S5**. Structural similarity as RMSD values calculated for the same protein with and without bound calcium; for clusters I and II separately and cluster I and cluster II together (I/II). **Table S6**. Values of distances and angles for the interactions shown in Figure 3 for the interaction of cluster I with the central β sheet. All designations are as in [63]. **Table S7**. Five classes of EF-Hand domains, based on the characteristics and conformational changes within the Clusters I and II (local level conformational changes) and within the entire EF-hand domain (global, domain level, conformational changes).
(DOC)
