# Supplementary material for: Two Structural Motifs within Canonical EF-Hand Calcium-Binding Domains Identify Five Different Classes of Calcium Buffers and Sensors
Source: PLoS One. 2014 Oct 14;9(10):e109287. doi: 10.1371/journal.pone.0109287 (PMC4196763; doi:10.1371/journal.pone.0109287)
Supplement: File S1 — Contains Tables S1–S7. Table S1. Assignment of interatomic contacts among the residues in clusters I and II according to the Interatomic Contacts software [61]. The “+” and “−” signs show presence or absence of Stabilizing and Destabilizing contacts, respectively, as defined by the Interatomic Contacts program. NI, absence of any interactions between the amino acids. 1EXR and 1IJ5 contain two EF-hand domains within chain A, while 1K94 contains two EF-hand domains within chain A, and one additional domain, shared between chains A and B. Table S2. Values of distances and angles for the CH-π interactions shown in Figure 2 for the interactions within cluster I. All designations are as in [62]. Table S3. Values of distances and angles for the CH-O interactions shown in Figure 2 for the interactions within cluster I. All designations are as in [63]. Table S4. Summarized areas of interacting surfaces among the amino acids within cluster I and cluster II (Figure 1), and the interacting area between the two clusters (I/II), for all EF-hand domains with known apo- and holo-form structures. NI, no interaction between the clusters. Table S5. Structural similarity as RMSD values calculated for the same protein with and without bound calcium; for clusters I and II separately and cluster I and cluster II together (I/II). Table S6. Values of distances and angles for the interactions shown in Figure 3 for the interaction of cluster I with the central β sheet. All designations are as in [63]. Table S7. Five classes of EF-Hand domains, based on the characteristics and conformational changes within the Clusters I and II (local level conformational changes) and within the entire EF-hand domain (global, domain level, conformational changes). (DOC) [file pone.0109287.s001.doc]

**Supporting Information**.

Table S1. Assignment of interatomic contacts among the residues in clusters I and II according to the Interatomic Contacts software [61]. The “+” and “-” signs show presence or absence of Stabilizing and Destabilizing contacts, respectively, as defined by the Interatomic Contacts program. NI, absence of any interactions between the amino acids. 1EXR and 1IJ5 contain two EF-hand domains within chain A, while 1K94 contains two EF-hand domains within chain A, and one additional domain, shared between chains A and B.

| PDB code | Aromatic Cluster I | | | Mixed Cluster II | | |
| --- | --- | --- | --- | --- | --- | --- |
|  | Contacting Residues: | Contacts: | | Contacting Residues: | Contacts: | |
|  |  | Stab. | Destab. |  | Stab. | Destab. |
| 1IG5 (A) | Phe10-Phe63 | + | - | Phe50-Lys25 | + | + |
|  | Phe10-Phe66 | + | - | Phe50-Leu28 | + | - |
| 1PSR (A) | Phe16-Phe71 | + | - | Phe58-Lys28 | + | - |
|  | Phe16-Phe74 | + | - | Phe58-Leu31 | + | - |
| 1K9U (A, B) | Phe9_A-Phe57_B | + | - | Met44_B-Leu22_A | + | + |
|  | Phe9_A-Phe60_B | + | - | Met44_B-Leu25_A | + | + |
| 1SRA (A) | Phe218-Leu266 | + | - | Phe253-His232 | NI | NI |
|  | Phe218-Trp269 | + | + | Phe253-Leu235 | + | - |
| 2PVB (A) | Phe47-Val99 | + | - | Leu86-Glu60 | + | + |
|  | Phe47-Phe102 | + | - | Leu86-Leu63 | + | + |
| 1EXR (A) | Phe16-Phe65 | + | - | Ile52-Thr29 | + | + |
|  | Phe16-Phe68 | + | - | Ile52-Leu32 | + | - |
|  | Phe89-Tyr138 | + | - | Ile125-Ala102 | + | + |
|  | Phe89-Phe141 | + | - | Ile125-Leu105 | + | - |
| 1F8H (A) | Phe21-Arg67 | + | - | Trp54-Gly33 | - | - |
|  | Phe21-Phe70 | + | - | Trp54-Val36 | + | - |
| 1IJ5 (A) | Phe226-Phe274 | + | - | Phe261-Arg239 | + | + |
|  | Phe226-Tyr277 | + | - | Phe261-Phe242 | + | - |
|  | Tyr291-Tyr341 | + | - | Phe328-Lys304 | + | - |
|  | Tyr291-Phe344 | + | - | Phe328-Val307 | + | - |
| 1K94 (A, B) | Phe58_A-Phe111_A | + | - | Ile98_A-Ala70_A | + | + |
|  | Phe58_A-Phe114_A | + | - | Ile98_A-Leu73_A | + | - |
|  | Phe128_A-Phe175_A | + | - | Val164_A-His141_A | + | + |
|  | Phe128_A-Tyr178_A | + | - | Val164_A-Leu144_A | + | - |
|  | Phe192_A-Tyr507_B | + | - | Phe492_B-Tyr207_A | + | - |
|  | Phe192_A-Phe510_B | + | - | Phe492_B-Phe210_A | + | - |
| 3BUX (B) | Trp190-Val238 | + | + | Lys225-Trp202 | + | + |
|  | Trp190-Phe241 | + | - | Lys225-Phe205 | NI | NI |
| 1WLM (A) | Phe21-Tyr79 | + | - | Phe66-Gly39 | - | - |
|  | Phe21-Phe82 | + | - | Phe66-Trp42 | + | + |

Table S2. Values of distances and angles for the CH-π interactions shown in Figure 2 for the interactions within cluster I. All designations are as in [62].

| PDB code | Contact | C-X (Å) | H-X (Å) | < CHX (degrees) | d_Hp-X_ (Å) | Contact | C-X (Å) | H-X (Å) | < CHX (degrees) | d_Hp-X_ (Å) |
| --- | --- | --- | --- | --- | --- | --- | --- | --- | --- | --- |
| 1IG5_A | CB/Phe63-π/Phe10 | 4.0 | 3.3 | 124 | 1.1 | CD2/Phe10-π/Phe66 | 4.1 | 3.0 | 168 | 1.7 |
| 1PSR_A | CD1/Phe71-π/Phe16 | 3.8 | 2.7 | 176 | 0.8 | CD1/Phe16-π/Phe74 | 3.8 | 2.9 | 140 | 1.4 |
| 1K9U_A,B | CD1/Phe57_B-π/Phe9_A | 3.9 | 3.0 | 140 | 1.0 | CD1/Phe9_A-π/Phe60_B | 4.3 | 3.3 | 159 | 1.7 |
| 1K9U_A,B | CD1/Phe57_A-π/Phe9_B | 3.6 | 3.0 | 115 | 1.5 | CD1/Phe9_B-π/Phe60_A | 4.0 | 2.9 | 162 | 1.3 |
| 1SRA_A | CD1/Leu266-π/Phe218 | 4.0 | 3.3 | 121 | 1.5 | CD2/Phe218-π/Trp269 | 3.9 | 2.9 | 152 | 1.2 |
| 2PVB_A | CA/Val99-π/Phe47 | 3.6 | 2.8 | 127 | 0.9 | CA/Phe47-π/Phe102 | 4.0 | 3.2 | 129 | 0.9 |
| 1EXR_A | CB/Phe65-π/Phe16 | 4.3 | 3.5 | 129 | 0.6 | CD1/Phe16-π/Phe68 | 4.0 | 3.0 | 162 | 1.7 |
| 1EXR_A | CB/Tyr138-π/Phe89 | 4.1 | 3.4 | 129 | 0.4 | CA/Phe89-π/Phe141 | 4.3 | 3.6 | 124 | 1.0 |
| 1F8H_A | CB/Arg67-π/Phe21 | 4.0 | 3.2 | 128 | 1.2 | CA/Phe21-π/Phe70 | 3.6 | 2.9 | 124 | 0.4 |
| 1IJ5_A | CB/Phe274-π/Phe226 | 4.1 | 3.4 | 124 | 0.8 | CD1/Phe226-π/Tyr277 | 3.7 | 2.7 | 178 | 1.1 |
| 1IJ5_A | CA/Tyr341-π/Tyr291 | 4.0 | 3.3 | 122 | 1.1 | CD1/Phe291-π/Phe344 | 4.4 | 3.3 | 169 | 1.8 |
| 1K94_A,B | CD1/Phe111_A-π/Phe58_A | 4.0 | 2.9 | 171 | 1.2 | CD1/Phe58_A-π/Phe114_A | 3.8 | 3.0 | 130 | 0.5 |
| 1K94_A,B | CD1/Phe175_A-π/Phe128_A | 3.7 | 2.7 | 159 | 0.4 | CD1/Phe128_A-π/Tyr178_A | 3.5 | 2.5 | 159 | 0.6 |
| 1K94_A,B | CD2/Tyr507_B-π/Phe192_A | 3.8 | 2.8 | 161 | 0.5 | CD2/Phe192_A-π/Phe510_B | 3.7 | 2.7 | 166 | 1.0 |
| 3BUX_B | CG2/Val238-π/Trp190 | 3.9 | 3.0 | 138 | 0.3 | CE3/Trp190-π/Phe241 | 3.8 | 2.9 | 142 | 0.8 |
| 1WLM_A | CD1/Tyr79-π/Phe21 | 4.2 | 3.2 | 149 | 1.0 | CD1/Phe21-π/Phe82 | 3.5 | 2.9 | 120 | 0.7 |

Table S3. Values of distances and angles for the CH-O interactions shown in Figure 2 for the interactions within cluster I. All designations are as in [63].

| PDB code | Contact | C-O (Å) | H-O (Å) | < CHO (degrees) | |θ| (elevation angle, degrees) |
| --- | --- | --- | --- | --- | --- |
| 1IG5_A | CB/Phe66-O/Phe63 | 3.4 | 2.5 | 140 | 41 |
| 1PSR_A | CB/Phe74-O/Phe71 | 3.4 | 2.5 | 145 | 39 |
| 1K9U_A,B | CB/Phe60_B-O/Phe57_B | 3.6 | 2.7 | 136 | 34 |
| 1K9U_A,B | CB/Phe60_A-O/Phe57_A | 3.4 | 2.4 | 140 | 29 |
| 1SRA_A | CB/Trp269-O/Leu266 | 3.5 | 2.5 | 145 | 42 |
| 2PVB_A | CB/Phe102-O/Val99 | 3.5 | 2.5 | 144 | 34 |
| 1EXR_A | CB/Phe68-O/Phe65 | 3.6 | 2.7 | 137 | 30 |
| 1EXR_A | CB/Phe141-O/Tyr138 | 3.4 | 2.5 | 142 | 31 |
| 1F8H_A | CB/Phe70-O/Arg67 | 4.3 | 3.4 | 140 | 59 |
| 1IJ5_A | CB/Tyr277-O/Phe274 | 3.0 | 2.0 | 149 | 29 |
| 1IJ5_A | CB/Phe344-O/Tyr341 | 3.7 | 2.8 | 141 | 35 |
| 1K94_A,B | CB/Phe114_A-O/Phe111_A | 3.4 | 2.4 | 140 | 31 |
| 1K94_A,B | CB/Tyr178_A-O/Phe175_A | 3.4 | 2.5 | 144 | 41 |
| 1K94_A,B | CB/Phe510_B-O/Tyr507_B | 3.4 | 2.4 | 147 | 30 |
| 3BUX_B | CB/Phe241-O/Val238 | 3.5 | 2.5 | 149 | 43 |
| 1WLM_A | CB/Phe82-O/Tyr79 | 3.2 | 2.2 | 146 | 30 |

Table S4. Summarized areas of interacting surfaces among the amino acids within cluster I and cluster II (Figure 1), and the interacting area between the two clusters (I/II), for all EF-hand domains with known *apo*- and *holo*-form structures. NI, no interaction between the clusters.

| PDB code | Ligand | Target | Cluster I | Surf. | Cluster II | Surf. | I/II surf. | Refs.* |
| --- | --- | --- | --- | --- | --- | --- | --- | --- |
| Calbindin D9K | | | | | | | | |
| 1CLB_A | - | - | F10, F63, F66 | 85.6 | F50, K25, L28 | 61.6 | 2.4 | [1] |
| 1B1G_A | 2 Ca^2+^ | - | F10, F63, F66 | 90.8 | F50, K25, L28 | 96.0 | 7.8 | [2] |
| S100A1 | | | | | | | | |
| 1K2H_A | - | - | F15, F71, F74 | 76.1 | M58, K30, L33 | 7.5 | 8.1 | [3] |
| 1ZFS_A | 2 Ca^2+^ | - | F15, F71, F74 | 102.2 | M58, K30, L33 | 77.4 | 32.6 | [4] |
| 2KBM_A | 2 Ca^2+^ | Capz Alpha-2 | F15, F71, F74 | 86.3 | M58, K30, L33 | 99.5 | 0.4 | [5] |
| 2K2F_A | 2 Ca^2+^ | RyRP12 | F15, F71, F74 | 76.3 | M58, K30, L33 | 79.4 | 22.9 | [6] |
| S100A4 | | | | | | | | |
| 1M31_A | - | - | F16, F72, Y75 | 63.3 | M59, K31, L34 | 27.5 | 23.1 | [7] |
| 3C1V_A | 2 Ca^2+^ | - | F16, F72, Y75 | 106.7 | M59, K31, L34 | 71.2 | 0.8 | [8] |
| 4ETO_A | 2 Ca^2+^ | Myosin-9 | F16, F72, Y75 | 97.3 | M59, K31, L34 | 79.3 | 1.9 | [9] |
| S100A5 | | | | | | | | |
| 2KAX_A | - | - | F16, F69, Y72 | 103.8 | M56, R31, L34 | 0.3 | 47.5 | [10] |
| 2KAY_A | 2 Ca^2+^ | - | F16, F69, Y72 | 122.0 | M56, R31, L34 | 81.0 | 6.1 | [10] |
| S100A6 | | | | | | | | |
| 2CNP_A | - | - | F16, F70, Y73 | 93.6 | M57, K31, L34 | 1.6 | 17.1 | [11] |
| 1JWD_A | 2 Ca^2+^ | - | F16, F70, Y73 | 83.5 | M57, K31, L34 | 64.9 | 9.3 | [12] |
| 2JTT_A | 2 Ca^2+^ | Cacybp | F16, F70, Y73 | 83.9 | M57, K31, L34 | 62.1 | 15.4 | [13] |
| S100B | | | | | | | | |
| 1B4C_A | - | - | F14, F70, F73 | 107.5 | M57, K29, L32 | 7.4 | 42.4 | [14] |
| 2K7O_A | 2 Ca^2+^ | - | F14, F70, F73 | 96.8 | M57, K29, L32 | 67.4 | 2.6 | [15] |
| 1MWN_A | 2 Ca^2+^ | Capz Alpha-1 | F14, F70, F73 | 76.5 | M57, K29, L32 | 79.6 | 21.3 | [16] |
| 1DT7_A | 2 Ca^2+^ | Antigen p53 | F14, F70, F73 | 88.7 | M57, K29, L32 | 49.8 | 31.4 | [17] |
| S100A13 | | | | | | | | |
| 1YUR_A | - | - | F20, F73, Y76 | 90.4 | M60, V35, F38 | 10.2 | 32.7 | [18] |
| 1YUT_A | 2 Ca^2+^ | - | F20, F73, Y76 | 102.4 | M60, V35, F38 | 34.5 | 30.3 | [18] |
| 2LE9_B | - | Ragec2 | F20, F73, Y76 | 39.0 | M60, V35, F38 | 83.7 | 4.8 | [19] |
| 2L5X_B | - | IL-1 Alpha | F20, F73, Y76 | 31.6 | M60, V35, F38 | 66.7 | 15.1 | [20] |
| 2K8M_B | - | C2a | F20, F73, Y76 | 84.7 | M60, V35, F38 | 29.1 | 29.6 | [21] |
| 2KI4_B | - | Hbgf-1 | F20, F73, Y76 | 97.4 | M60, V35, F38 | 31.3 | 30.2 | [22] |
| 2KI6_C | - | Hbgf-1, C2a | F20, F73, Y76 | 97.4 | M60, V35, F38 | 31.3 | 30.2 | [22] |
| S100A16 | | | | | | | | |
| 2L50_A | - | - | F19, F76, Y79 | 108.8 | I63, K35, F38 | 86.9 | 34.1 | [23] |
| 2L51_A | 2 Ca^2+^ | - | F19, F76, Y79 | 91.5 | I63, K35, F38 | 79.3 | 38.8 | [23] |
| S100P | | | | | | | | |
| 1OZO_A | - | - | F15, F71, F74 | 84.7 | L58, K30, L33 | 63.6 | 0.8 | [24] |
| 1J55_A | 2 Ca^2+^ | - | F15, F71, F74 | 95.5 | L58, K30, L33 | 57.7 | 3.2 | [25] |
| Polcalcin | | | | | | | | |
| 2LVI_A | - | - | F8, F56, F59 | 65.5 | M43, L21, L24 | 65.7 | 17.2 | [26] |
| 2LVK_A | 2 Ca^2+^ | - | F8, F56, F59 | 99.2 | M43, L21, L24 | 57.7 | 3.3 | [26] |
| Parvalbumin | | | | | | | | |
| 2JWW_A | - | - | F47, V99, F102 | 80.4 | M86, E60, L63 | 67.8 | NI | [27] |
| 1RWY_A | 2 Ca^2+^ | - | F47, V99, F102 | 78.6 | M86, E60, L63 | 66.3 | NI | [28] |
| Oncomodulin | | | | | | | | |
| 2NLN_A | - | - | F47, A99, F102 | 75.6 | M86, G60, L63 | 59.2 | NI | [29] |
| 1RRO_A | 2 Ca^2+^ | - | F47, A99, F102 | 71.2 | M86, G60, L63 | 47.6 | NI | [30] |

Table S4 (Continued).

| PDB code | Ligand | Target | Cluster I | Surf. | Cluster II | Surf. | I/II surf. | Refs.* |
| --- | --- | --- | --- | --- | --- | --- | --- | --- |
| Calmodulin, N-domain | | | | | | | | |
| 1QX5_R | - | - | F16, F65, F68 | 82.3 | I52, T29, L32 | 89.8 | 32.7 | [31] |
| 3CLN_A | 2 Ca^2+^ | - | F16, F65, F68 | 79.6 | I52, T29, L32 | 64.2 | NI | [32] |
| 1QX7_R | - | Sk2 | F16, F65, F68 | 79.1 | I52, T29, L32 | 82.8 | 25.8 | [31] |
| 1G4Y_R | 2 Ca^2+^ | Rsk2 | F16, F65, F68 | 82.4 | I52, T29, L32 | 49.7 | NI | [33] |
| 4EHQ_A | 2 Ca^2+^ | - | F16, F65, F68 | 85.3 | I52, T29, L32 | 54.5 | NI | [34] |
| 3BXK_A | 2 Ca^2+^ | Cav2.1 | F16, F65, F68 | 90.2 | I52, T29, L32 | 58.3 | NI | [35] |
| 3BXL_A | 2 Ca^2+^ | Cav2.3 | F16, F65, F68 | 86.9 | I52, T29, L32 | 42.0 | NI | [35] |
| 2HQW_A | 2 Ca^2+^ | NR1C1 | F16, F65, F68 | 76.9 | I52, T29, L32 | 39.7 | NI | [36] |
| 1NIW_A | 2 Ca^2+^ | NOS | F16, F65, F68 | 87.3 | I52, T29, L32 | 53.6 | 1.0 | [37] |
| 2YGG_B | 2 Ca^2+^ | NHE1 | F17, F66, F69 | 102.5 | I53, T30, L33 | 57.7 | NI | [38] |
| Calmodulin, C-domain | | | | | | | | |
| 1QX5_R, I | - | - | F89_I, Y138_R, F141_R | 77.0 | I125_R, A102_I, L105_I | 42.8 | 66.6 | [31] |
| 3CLN_A | 2 Ca^2+^ | - | F89, Y138, F141 | 80.3 | I125, A102, L105 | 32.5 | NI | [32] |
| 1QX7_R, I | - | Sk2 | F89_I, Y138_R, F141_R | 76.4 | I125_R, A102_I, L105_I | 42.8 | 50.3 | [31] |
| 1G4Y_R | - | - | F89, Y138, F141 | 67.0 | I125, A102, L105 | 65.0 | 12.4 | [33] |
| 4EHQ_A | 2 Ca^2+^ | Orai-1 | F89, Y138, F141 | 65.1 | I125, A102, L105 | 45.6 | NI | [34] |
| 3BXK_A | 2 Ca^2+^ | Cav2.1 | F89, Y138, F141 | 89.0 | I125, A102, L105 | 56.3 | NI | [35] |
| 3BXL_A | 2 Ca^2+^ | Cav2.3 | F89, Y138, F141 | 61.9 | I125, A102, L105 | 58.2 | NI | [35] |
| 2HQW_A | 2 Ca^2+^ | NR1C1 | F89, Y138, F141 | 69.8 | I125, A102, L105 | 48.5 | NI | [36] |
| 1NIW_A | 2 Ca^2+^ | NOS | F89, Y138, F141 | 85.3 | I125, A102, L105 | 37.5 | NI | [37] |
| 2YGG_B | 2 Ca^2+^ | NHE1 | F90, Y139, F142 | 73.5 | I126, A103, L106 | 58.2 | NI | [38] |
| Troponin C | | | | | | | | |
| 1TNP_A | - | - | F26, F75, F78 | 87.0 | I62, T39, L42 | 60.3 | 19.4 | [39] |
| 1TNQ_A | 2 Ca^2+^ | - | F26, F75, F78 | 104.2 | I62, T39, L42 | 76.5 | NI | [39] |
| 1YTZ_C | 2 Ca^2+^ | Troponin I, T | F25, F74, F77 | 81.2 | I61, T38, L41 | 56.1 | NI | [40] |
| Cbp40, N-domain | | | | | | | | |
| 1IJ5_A | - | - | F226, F274, Y277 | 89.1 | F261, R239, F242 | 104.1 | 0.4 | [41] |
| 1IJ6_A | 2 Ca^2+^ | - | F226, F274, Y277 | 82.5 | F261, R239, F242 | 107.1 | 0.6 | [41] |
| Cbp40, C-domain | | | | | | | | |
| 1IJ5_A | - | - | Y291, Y341, F344 | 62.1 | F238, K304, V307 | 67.7 | 28.7 | [41] |
| 1IJ6_A | 2 Ca^2+^ | - | Y291, Y341, F344 | 69.3 | F238, K304, V307 | 66.9 | 19.9 | [41] |
| Calpain, N-domain | | | | | | | | |
| 1AJ5_A | - | - | F107, F163, F166 | 94.6 | V150, A119, L122 | 43.6 | 31.5 | [42] |
| 1DVI_A | 2 Ca^2+^ | - | F107, F163, F166 | 75.7 | V150, A119, L122 | 49.3 | 21.6 | [42] |
| 1DF0_B | - | M-Calpain | F21, F77, F80 | 97.2 | V64, A33, L36 | 43.7 | 28.7 | [43] |
| 3DF0_B | 2 Ca^2+^ | M-Calpain, Calpastatin | F107, F163, F166 | 85.0 | V150, A119, L122 | 43.2 | 21.4 | [44] |
| 3BOW_B | 2 Ca^2+^ | M-Calpain, Calpastatin | F107, F163, F166 | 87.7 | V150, A119, L122 | 45.4 | 21.7 | [45] |
| Calpain, C-domain | | | | | | | | |
| 1AJ5_A | - | - | Y180, F228, F231 | 73.1 | I216, S193, L196 | 23.0 | 6.1 | [42] |
| 1DVI_A | 2 Ca^2+^ | - | Y180, F228, F231 | 81.1 | I216, S193, L196 | 64.7 | 1.4 | [42] |
| 1DF0_B | - | M-Calpain | Y94, F142, F145 | 72.5 | I130, S107, L110 | 66.1 | 10.6 | [43] |
| 3DF0_B | 2 Ca^2+^ | M-Calpain, Calpastatin | Y180, F228, F231 | 72.2 | I216, S193, L196 | 60.8 | 1.9 | [44] |
| 3BOW_B | 2 Ca^2+^ | M-Calpain, Calpastatin | Y180, F228, F231 | 69.3 | V150, A119, L122 | 56.1 | 0.2 | [45] |

* All (forty five) references in Table S4 are specific for the supplementary material only, and are given below in this file and not in the main body of the manuscript.

Table S5. Structural similarity as RMSD values calculated for the same protein with and without bound calcium; for clusters I and II separately and cluster I and cluster II together (I/II).

| PDB codes | Cluster I | RMSD | | Cluster II | RMSD | | I/II RMSD | |
| --- | --- | --- | --- | --- | --- | --- | --- | --- |
|  |  | Back bone | Heavy |  | Back bone | Heavy | Back bone | Heavy |
| Calbindin D9K | | | | | | | | |
| 1CLB_A-1B1G_A | F10, F63, F66 | 0.42 | 1.03 | F50, K25, L28 | 0.34 | 1.68 | 0.90 | 1.49 |
| S100A1 | | | | | | | | |
| 1K2H_A-1ZFS_A | F15, F71, F74 | 0.99 | 3.68 | M58, K30, L33 | 1.54 | 4.32 | 2.53 | 4.29 |
| 1K2H_A-2KBM_A | F15, F71, F74 | 0.41 | 3.45 | M58, K30, L33 | 1.52 | 4.27 | 2.13 | 4.13 |
| 1K2H_A-2K2F_A | F15, F71, F74 | 0.47 | 1.82 | M58, K30, L33 | 1.63 | 4.26 | 2.18 | 3.49 |
| S100A4 | | | | | | | | |
| 1M31_A-3C1V_A | F16, F72, Y75 | 0.81 | 1.63 | M59, K31, L34 | 1.56 | 3.44 | 2.60 | 3.49 |
| 1M31_A-4ETO_A | F16, F72, Y75 | 0.83 | 1.65 | M59, K31, L34 | 1.56 | 3.53 | 2.43 | 3.43 |
| S100A5 | | | | | | | | |
| 2KAX_A-2KAY_A | F16, F69, Y72 | 0.33 | 0.85 | M56, R31, L34 | 1.61 | 5.21 | 2.10 | 3.77 |
| S100A6 | | | | | | | | |
| 2CNP_A-1JWD_A | F16, F70, Y73 | 0.34 | 0.96 | M57, K31, L34 | 1.69 | 3.58 | 1.99 | 2.74 |
| 2CNP_A-2JTT_A | F16, F70, Y73 | 0.39 | 1.16 | M57, K31, L34 | 1.51 | 3.35 | 1.71 | 2.50 |
| S100B | | | | | | | | |
| 1B4C_A-2K7O_A | F14, F70, F73 | 0.38 | 1.20 | M57, K29, L32 | 1.50 | 3.37 | 1.99 | 2.95 |
| 1B4C_A-1MWN_A | F14, F70, F73 | 0.77 | 2.70 | M57, K29, L32 | 1.63 | 3.44 | 2.11 | 3.50 |
| 1B4C_A-1DT7_A | F14, F70, F73 | 0.86 | 1.72 | M57, K29, L32 | 1.61 | 3.09 | 2.03 | 2.89 |
| S100A13 | | | | | | | | |
| 1YUR_A-1YUT_A | F20, F73, Y76 | 1.33 | 1.91 | M60, V35, F38 | 1.72 | 3.88 | 2.45 | 3.11 |
| 1YUR_A-2LE9_B | F20, F73, Y76 | 1.55 | 2.64 | M60, V35, F38 | 1.76 | 3.89 | 2.82 | 3.59 |
| 1YUR_A-2L5X_B | F20, F73, Y76 | 1.68 | 2.63 | M60, V35, F38 | 1.71 | 3.83 | 2.87 | 3.50 |
| 1YUR_A-2K8M_B | F20, F73, Y76 | 1.29 | 2.22 | M60, V35, F38 | 1.73 | 3.81 | 2.54 | 3.30 |
| 1YUR_A-2KI4_B | F20, F73, Y76 | 1.24 | 1.86 | M60, V35, F38 | 1.68 | 3.82 | 2.42 | 3.07 |
| 1YUR_A-2KI6_C | F20, F73, Y76 | 1.24 | 1.86 | M60, V35, F38 | 1.68 | 3.82 | 2.42 | 3.07 |
| S100A16 | | | | | | | | |
| 2L50_A-2L51_A | F19, F76, Y79 | 0.63 | 2.62 | I63, K35, F38 | 0.94 | 1.92 | 1.34 | 2.64 |
| S100P | | | | | | | | |
| 1OZO_A-1J55_A | F15, F71, F74 | 0.53 | 2.01 | L58, K30, L33 | 0.36 | 0.98 | 0.94 | 1.78 |
| Polcalcin | | | | | | | | |
| 2LVI_A-2LVK_A | F8, F56, F59 | 0.50 | 1.60 | M43, L21, L24 | 1.70 | 3.05 | 1.92 | 2.55 |
| Parvalbumin | | | | | | | | |
| 2JWW_A-1RWY_A | F47, V99, F102 | 0.49 | 1.19 | M86, E60, L63 | 1.11 | 1.88 | 1.26 | 1.76 |
| Oncomodulin | | | | | | | | |
| 2NLN_A-1RRO_A | F47, A99, F102 | 0.58 | 1.25 | M86, G60, L63 | 1.67 | 1.89 | 2.02 | 2.11 |

Table S5 (Continues)

| Calmodulin, N-domain | | | | | | | | |
| --- | --- | --- | --- | --- | --- | --- | --- | --- |
| 1QX5_R-3CLN_A | F16, F65, F68 | 0.55 | 1.67 | I52, T29, L32 | 0.46 | 1.02 | 1.68 | 2.08 |
| 1QX5_R-1QX7_R | F16, F65, F68 | 0.19 | 0.89 | I52, T29, L32 | 0.29 | 0.47 | 0.33 | 0.76 |
| 1QX5_R-1G4Y_R | F16, F65, F68 | 0.49 | 0.70 | I52, T29, L32 | 0.67 | 1.13 | 1.62 | 1.67 |
| 1QX5_R-4EHQ_A | F16, F65, F68 | 0.67 | 1.24 | I52, T29, L32 | 0.38 | 0.94 | 1.65 | 1.89 |
| 1QX5_R-3BXK_A | F16, F65, F68 | 0.58 | 1.42 | I52, T29, L32 | 0.61 | 1.34 | 1.89 | 2.11 |
| 1QX5_R-3BXL_A | F16, F65, F68 | 0.64 | 1.42 | I52, T29, L32 | 0.55 | 1.30 | 1.82 | 2.06 |
| 1QX5_R-2HQW_A | F16, F65, F68 | 0.59 | 1.08 | I52, T29, L32 | 0.46 | 1.26 | 1.71 | 1.95 |
| 1QX5_R-1NIW_A | F16, F65, F68 | 0.65 | 1.20 | I52, T29, L32 | 0.60 | 1.07 | 1.22 | 1.56 |
| 1QX5_R-2YGG_B | F16/17, F65/66, F68/69 | 0.57 | 1.12 | I52/53, T29/30, L32/33 | 0.44 | 0.73 | 1.79 | 1.90 |
| Calmodulin, C-domain | | | | | | | | |
| 1QX5_R, I-3CLN_A | F89_I/89, Y138_R/138, F141_R/141 | 0.87 | 3.82 | I125_R/125, A102_I/102, L105_I/105 | 1.16 | 1.96 | 1.63 | 3.70 |
| 1QX5_R, I-1QX7_R, I | F89_I, Y138_R, F141_R | 0.19 | 0.21 | I125_R, A102_I, L105_I | 0.24 | 0.44 | 0.31 | 0.37 |
| 1QX5_R, I-1G4Y_R | F89_I/89, Y138_R/138, F141_R/141 | 0.87 | 2.08 | I125_R/125, A102_I/102, L105_I/105 | 0.68 | 0.86 | 1.37 | 2.00 |
| 1QX5_R, I-4EHQ_A | F89_I/89, Y138_R/138, F141_R/141 | 0.80 | 3.83 | I125_R/125, A102_I/102, L105_I/105 | 1.09 | 1.96 | 1.64 | 3.72 |
| 1QX5_R, I-3BXK_A | F89_I/89, Y138_R/138, F141_R/141 | 1.04 | 3.88 | I125_R/125, A102_I/102, L105_I/105 | 1.09 | 1.86 | 1.65 | 3.71 |
| 1QX5_R, I-3BXL_A | F89_I/89, Y138_R/138, F141_R/141 | 1.10 | 4.03 | I125_R/125, A102_I/102, L105_I/105 | 1.11 | 1.96 | 1.71 | 3.90 |
| 1QX5_R, I-2HQW_A | F89_I/89, Y138_R/138, F141_R/141 | 0.95 | 3.92 | I125_R/125, A102_I/102, L105_I/105 | 1.07 | 1.94 | 1.82 | 3.76 |
| 1QX5_R, I-1NIW_A | F89_I/89, Y138_R/138, F141_R/141 | 0.84 | 3.83 | I125_R/125, A102_I/102, L105_I/105 | 1.06 | 1.94 | 1.67 | 3.59 |
| 1QX5_R, I-2YGG_B | F89_I/90, Y138_R/139, F141_R/142 | 0.86 | 3.86 | I125_R/126, A102_I/103, L105_I/106 | 1.13 | 1.76 | 1.60 | 3.75 |
| Troponin C | | | | | | | | |
| 1TNP_A-1TNQ_A | F26, F75, F78 | 0.64 | 1.53 | I62, T39, L42 | 0.91 | 1.82 | 2.03 | 2.26 |
| 1TNP_A-1YTZ_C | F26/25, F75/74, F78/77 | 0.90 | 1.32 | I62/61, T39/38, L42/41 | 1.14 | 2.14 | 2.51 | 2.58 |
| Cbp40, N-domain | | | | | | | | |
| 1IJ5_A-1IJ6_A | F226, F274, Y277 | 0.13 | 0.30 | F261, R239, F242 | 0.27 | 0.55 | 0.25 | 0.44 |
| Cbp40, C-domain | | | | | | | | |
| 1IJ5_A-1IJ6_A | Y291, Y341, F344 | 0.13 | 0.19 | F328, K304, V307 | 0.16 | 1.05 | 0.24 | 0.71 |
| Calpain, N-domain | | | | | | | | |
| 1AJ5_A-1DVI_A | F107, F163, F166 | 1.05 | 2.04 | V150, A119, L122 | 0.14 | 0.18 | 1.16 | 1.89 |
| 1AJ5_A-1DF0_B | F107/21, F163/77, F166/80 | 0.28 | 0.44 | V150/64, A119/33, L122/36 | 0.12 | 0.12 | 0.29 | 0.39 |
| 1AJ5_A-3DF0_B | F107, F163, F166 | 1.09 | 1.78 | V150, A119, L122 | 0.15 | 0.24 | 1.37 | 1.82 |
| 1AJ5_A-3BOW_B | F107, F163, F166 | 1.02 | 1.76 | V150, A119, L122 | 0.16 | 0.22 | 1.23 | 1.71 |

Table S5 (Continued)

| Calpain, C-domain | | | | | | | | |
| --- | --- | --- | --- | --- | --- | --- | --- | --- |
| 1AJ5_A-1DVI_A | Y180, F228, F231 | 0.40 | 1.01 | I216, S193, L196 | 0.81 | 1.46 | 0.70 | 1.24 |
| 1AJ5_A-1DF0_B | Y180/94, F228/142, F231/145 | 0.14 | 0.55 | I216/130, S193/107, L196/110 | 0.71 | 0.88 | 0.54 | 0.70 |
| 1AJ5_A-3DF0_B | Y180, F228, F231 | 0.59 | 1.11 | I216, S193, L196 | 0.81 | 1.43 | 0.75 | 1.28 |
| 1AJ5_A-3BOW_B | Y180, F228, F231 | 0.47 | 1.07 | I216, S193, L196 | 0.84 | 1.59 | 0.74 | 1.31 |

Table S6. Values of distances and angles for the interactions shown in Figure 3 for the interaction of cluster I with the central β sheet. All designations are as in [63].

| PDB code | Contact | C-O (Å) | H-O (Å) | < CHO (deg.) | |θ| (elevation angle, deg.) | Contact | C-O (Å) | H-O (Å) | < CHO (deg.) | |θ| (elevation angle, deg.) |
| --- | --- | --- | --- | --- | --- | --- | --- | --- | --- | --- |
| 1IG5_A | CZ/Phe10-O/Asn21 | 3.6 | 2.8 | 131 | 58 | CZ/Phe10-O/Val61 | 3.7 | 2.9 | 132 | 11 |
| 1PSR_A | CZ/Phe16-O/Asp24 | 3.3 | 2.5 | 124 | 31 | CE1/Phe16-O/Ile69 | 3.9 | 3.1 | 133 | 57 |
| 1K9U_A,B | CZ/Phe9_A-O/Gly18_A | 3.3 | 2.5 | 129 | 48 | CE1/Phe9_A-O/Ile55_B | 3.6 | 2.9 | 124 | 62 |
| 1K9U_A,B | CZ/Phe9_B-O/Gly18_B | 3.5 | 2.5 | 154 | 78 | CE1/Phe9_B-O/Ile55_A | 4.1 | 3.1 | 144 | 47 |
| 1SRA_A | CZ/Phe218-O/Gly228 | 3.3 | 2.4 | 149 | 74 | CE2/Phe218-O/Ile264 | 3.6 | 2.8 | 138 | 53 |
| 2PVB_A | CZ/Phe47-O/Gly56 | 3.5 | 2.6 | 135 | 52 | CE1/Phe47-O/Ile97 | 3.4 | 2.5 | 138 | 58 |
| 1EXR_A | CZ/Phe16-O/Gly25 | 3.6 | 2.7 | 142 | 68 | CE1/Phe16-O/Ile63 | 3.7 | 3.0 | 125 | 63 |
| 1EXR_A | CZ/Phe89-O/Gly98 | 3.5 | 2.5 | 149 | 80 | CE1/Phe89-O/Ile136 | 3.7 | 2.9 | 131 | 60 |
| 1F8H_A | CE2/Phe21-O/Gly29 | 4.1 | 3.2 | 145 | 69 | CD2/Phe21-O/Leu65 | 3.5 | 2.7 | 132 | 27 |
| 1IJ5_A | CZ/Phe226-O/Gly235 | 3.9 | 3.2 | 119 | 59 | CZ/Phe226-O/Val272 | 4.2 | 3.4 | 135 | 21 |
| 1IJ5_A | CE2/Tyr291-O/Gly300 | 3.7 | 3.0 | 125 | 36 | OH/Tyr291-O/Leu339 | 3.0 | NA | NA | NA |
| 1K94_A,B | CE2/Phe58_A-O/Gly66_A | 3.9 | 3.0 | 143 | 50 | CZ/Phe58_A-O/Met109_A | 3.7 | 2.8 | 149 | 26 |
| 1K94_A,B | CZ/Phe128_A-O/Gly137_A | 3.5 | 2.5 | 154 | 71 | CE1/Phe128_A-O/Ile173_A | 4.5 | 3.6 | 142 | 37 |
| 1K94_A,B | CZ/Phe192_A-O/Gly201_A | 4.2 | 3.4 | 134 | 49 | CZ/Phe192_A-O/Phe505_B | 3.6 | 2.8 | 133 | 32 |
| 3BUX_B | CZ2/Trp190-O/Thr198 | 4.2 | 3.4 | 132 | 76 | CH2/Trp190-O/Ile236 | 3.6 | 2.5 | 171 | 53 |
| 1WLM_A | CZ/Phe21-O/Gln35 | 4.6 | 3.7 | 137 | 66 | CZ/Phe21-O/Ile77 | 4.5 | 3.5 | 154 | 36 |

NA, not applicable in this case.

Table S7. Five classes of EF-Hand domains, based on the characteristics and conformational changes within the Clusters I and II (local level conformational changes) and within the entire EF-hand domain (global, domain level, conformational changes).

| Domain Class | Changes upon calcium binding | Representative Structures | PDB Code: | | UniProtKB Reference: |
| --- | --- | --- | --- | --- | --- |
|  |  |  | Apo form | Holo form |  |
| Open Static: (open domain conformation and no any type of conformational changes) | Cluster I conformation does not change; Cluster II conformation does not change; Cluster I/Cluster II relative conformation does not rearrange; Domain conformation remains open. | Calbindin 9K, S100P, parvalbumin, oncomodulin, Cbp40 (N-domain) | 1CLB_A | 1B1G_A | P02633 |
|  |  |  | 1OZO_A | 1J55_A | P25815 |
|  |  |  | 2JWW_A | 1RWY_A | P02625 |
|  |  |  | 2NLN_A | 1RRO_A | P02631 |
|  |  |  | 1IJ5_A | 1IJ6_A | P14725 |
| Closed Static: (closed domain conformation and no any type of conformational changes) | Cluster I conformation does not change; Cluster II conformation does not change; Cluster I/Cluster II relative conformation does not rearrange; Domain conformation remains closed. | S100A16, Cbp40 (C-domain), calpain (N-domain) | 2L50_A | 2L51_A | Q96FQ6 |
|  |  |  | 1IJ5_A | 1IJ6_A | P14725 |
|  |  |  | 1AJ5_A | 1DVI_A | Q64537 |
| Local Dynamic: (simultaneous local and global conformational changes) | Cluster I conformation does not change; Cluster II conformation changes to more compact; Cluster I/Cluster II relative conformation rearranges; Domain conformation changes from closed to open. | S100A1, S100A4, S100A5, S100A6, S100B | 1K2H_A | 1ZFS_A | P35467 |
|  |  |  | 1M31_A | 3C1V_A | P26447 |
|  |  |  | 2KAX_A | 2KAY_A | P33763 |
|  |  |  | 2CNP_A | 1JWD_A | P30801 |
|  |  |  | 1B4C_A | 2K7O_A | P04631 |
| Dynamic: (only global conformational changes) | Cluster I conformation does not change; Cluster II conformation does not change; Cluster I/Cluster II relative conformation rearranges; Domain conformation changes from closed to open. | Polcalcin, calmodulin (N-domain), calmodulin (C-domain), troponin C | 2LVI_A | 2LVK_A | O82040 |
|  |  |  | 1QX5_R | 3CLN_A | P62161 |
|  |  |  | 1QX5_R, I | 3CLN_A | P62161 |
|  |  |  | 1TNP_A | 1TNQ_A | P02588 |
| Local Static: (open domain conformation and only local conformational changes) | Cluster I conformation does not change; Cluster II conformation changes to more compact; Cluster I/Cluster II relative conformation does not rearrange; Domain conformation remains open. | S100A13, calpain (C-domain) | 1YUR_A, | 1YUT_A | Q99584 |
|  |  |  | 1AJ5_A | 1DVI_A | Q64537 |

**References** (Table S4)

1. Skelton NJ, Kördel J, Chazin WJ (1995) Determination of the solution structure of Apo calbindin D9k by NMR spectroscopy. J Mol Biol 249: 441-462.

2. Kördel J, Pearlman DA, Chazin WJ (1997) Protein solution structure calculations in solution: solvated molecular dynamics refinement of calbindin D9k. J Biomol NMR 10: 231-243.

3. Rustandi RR, Baldisseri DM, Inman KG, Nizner P, Hamilton SM, et al. (2002) Three-dimensional solution structure of the calcium-signaling protein apo-S100A1 as determined by NMR. Biochemistry 41: 788-796.

4. Wright NT, Varney KM, Ellis KC, Markowitz J, Gitti RK, et al. (2005) The three-dimensional solution structure of Ca(2+)-bound S100A1 as determined by NMR spectroscopy. J Mol Biol 353: 410-426.

5. Wright NT, Cannon BR, Wilder PT, Morgan MT, Varney KM, et al. (2009) Solution structure of S100A1 bound to the CapZ peptide (TRTK12). J Mol Biol 386: 1265-1277.

6. Wright NT, Prosser BL, Varney KM, Zimmer DB, Schneider MF, et al. (2008) S100A1 and calmodulin compete for the same binding site on ryanodine receptor. J Biol Chem 283: 26676-26683.

7. Vallely KM, Rustandi RR, Ellis KC, Varlamova O, Bresnick AR, et al. (2002) Solution structure of human Mts1 (S100A4) as determined by NMR spectroscopy. Biochemistry 41: 12670-12680.

8. Gingras AR, Basran J, Prescott A, Kriajevska M, Bagshaw CR, et al. (2008) Crystal structure of the Ca(2+)-form and Ca(2+)-binding kinetics of metastasis-associated protein, S100A4. FEBS Lett 582: 1651-1656.

9. Ramagopa UA, Dulyaninova NG, Kumar PR, Almo SC, Bresnick AR. Structure of S100A4 with bound peptide P. To be Published.

10. Bertini I, Das Gupta S, Hu X, Karavelas T, Luchinat C, et al. (2009) Solution structure and dynamics of S100A5 in the apo and Ca2+-bound states. J Biol Inorg Chem 14: 1097-1107.

11. Mäler L, Potts BC, Chazin WJ (1999) High resolution solution structure of apo calcyclin and structural variations in the S100 family of calcium-binding proteins. J Biomol NMR 13: 233-247.

12. Mäler L, Sastry M, Chazin WJ (2002) A structural basis for S100 protein specificity derived from comparative analysis of apo and Ca(2+)-calcyclin. J Mol Biol 317: 279-290.

13. Lee YT, Dimitrova YN, Schneider G, Ridenour WB, Bhattacharya S, et al. (2008) Structure of the S100A6 complex with a fragment from the C-terminal domain of Siah-1 interacting protein: a novel mode for S100 protein target recognition. Biochemistry 47: 10921-10932.

14. Drohat AC, Tjandra N, Baldisseri DM, Weber DJ (1999) The use of dipolar couplings for determining the solution structure of rat apo-S100B(betabeta). Protein Sci 8: 800-809.

15. Wright NT, Inman KG, Levine JA, Cannon BR, Varney KM, et al. (2008) Refinement of the solution structure and dynamic properties of Ca(2+)-bound rat S100B. J Biomol NMR 42: 279-286.

16. Inman KG, Yang R, Rustandi RR, Miller KE, Baldisseri DM, et al. (2002) Solution NMR structure of S100B bound to the high-affinity target peptide TRTK-12. J Mol Biol 324: 1003-1014.

17. Rustandi RR, Baldisseri DM, Weber DJ (2000) Structure of the negative regulatory domain of p53 bound to S100B(betabeta). Nat Struct Biol 7: 570-574.

18. Arnesano F, Banci L, Bertini I, Fantoni A, Tenori L, et al. (2005) Structural interplay between calcium(II) and copper(II) binding to S100A13 protein. Angew Chem Int Ed Engl 44: 6341-6344.

19. Rani SG, Sepuru KM, Yu C (2014) Interaction of S100A13 with C2 domain of receptor for advanced glycation end products (RAGE). Biochim Biophys Acta 1844: 1718-1728.

20. Mohan SK, Yu C (2011) The IL1alpha-S100A13 heterotetrameric complex structure: a component in the non-classical pathway for interleukin 1alpha secretion. J Biol Chem 286: 14608-14617.

21. Mohan SK, Rani SG, Kumar SM, Yu C (2009) S100A13-C2A binary complex structure-a key component in the acidic fibroblast growth factor for the non-classical pathway. Biochem Biophys Res Commun 380: 514-519.

22. Mohan SK, Rani SG, Yu C (2010) The heterohexameric complex structure, a component in the non-classical pathway for fibroblast growth factor 1 (FGF1) secretion. J Biol Chem 285: 15464-15475.

23. Babini E, Bertini I, Borsi V, Calderone V, Hu X, et al. (2011) Structural characterization of human S100A16, a low-affinity calcium binder. J Biol Inorg Chem 16: 243-256.

24. Lee YC, Volk DE, Thiviyanathan V, Kleerekoper Q, Gribenko AV, et al. (2004) NMR structure of the Apo-S100P protein. J Biomol NMR 29: 399-402.

25. Zhang H, Wang G, Ding Y, Wang Z, Barraclough R, et al. (2003) The crystal structure at 2Å resolution of the Ca2+ -binding protein S100P. J Mol Biol 325: 785-794.

26. Henzl MT, Sirianni AG, Wycoff WG, Tan A, Tanner JJ (2013) Solution structures of polcalcin Phl p 7 in three ligation states: Apo-, hemi-Mg2+-bound, and fully Ca2+-bound. Proteins 81:300-315.

27. Henzl MT, Tanner JJ (2008) Solution structure of Ca2+-free rat alpha-parvalbumin. Protein Sci 17: 431-438.

28. Bottoms CA, Schuermann JP, Agah S, Henzl MT, Tanner JJ (2004) Crystal structure of rat alpha-parvalbumin at 1.05 Å resolution. Protein Sci 13: 1724-1734.

29. Henzl MT, Tanner JJ (2007) Solution structure of Ca2+-free rat beta-parvalbumin (oncomodulin). Protein Sci 16: 1914-1926.

30. Ahmed FR, Rose DR, Evans SV, Pippy ME, To R (1993) Refinement of recombinant oncomodulin at 1.30 Å resolution. J Mol Biol 230: 1216-1224.

31. Schumacher MA, Crum M, Miller MC (2004) Crystal structures of apocalmodulin and an apocalmodulin/SK potassium channel gating domain complex. Structure 12: 849-860.

32. Babu YS, Bugg CE, Cook WJ (1988) Structure of calmodulin refined at 2.2 Å resolution. J Mol Biol 204:191-204.

33. Schumacher MA, Rivard AF, Bächinger HP, Adelman JP (2001) Structure of the gating domain of a Ca2+-activated K+ channel complexed with Ca2+/calmodulin. Nature 410: 1120-1124.

34. Liu Y, Zheng X, Mueller GA, Sobhany M, DeRose EF, et al. (2012) Crystal structure of calmodulin binding domain of orai1 in complex with Ca2+ calmodulin displays a unique binding mode. J Biol Chem 287: 43030-43041.

35. Mori MX, Vander Kooi CW, Leahy DJ, Yue DT (2008) Crystal structure of the CaV2 IQ domain in complex with Ca2+/calmodulin: high-resolution mechanistic implications for channel regulation by Ca2+. Structure 16: 607-620.

36. Ataman ZA, Gakhar L, Sorensen BR, Hell JW, Shea MA (2007) The NMDA receptor NR1 C1 region bound to calmodulin: structural insights into functional differences between homologous domains. Structure 15: 1603-1617.

37. Aoyagi M, Arvai AS, Tainer JA, Getzoff ED (2003) Structural basis for endothelial nitric oxide synthase binding to calmodulin. EMBO J 22: 766-775.

38. Köster S, Pavkov-Keller T, Kühlbrandt W, Yildiz Ö (2011) Structure of human Na+/H+ exchanger NHE1 regulatory region in complex with calmodulin and Ca2+. J Biol Chem 286: 40954-40961.

39. Gagné SM, Tsuda S, Li MX, Smillie LB, Sykes BD (1995) Structures of the troponin C regulatory domains in the apo and calcium-saturated states. Nat Struct Biol 2: 784-789.

40. Vinogradova MV, Stone DB, Malanina GG, Karatzaferi C, Cooke R, et al. (2005) Ca(2+)-regulated structural changes in troponin. Proc Natl Acad Sci USA 102: 5038-5043.

41. Iwasaki W, Sasaki H, Nakamura A, Kohama K, Tanokura M (2003) Metal-free and Ca2+-bound structures of a multidomain EF-hand protein, CBP40, from the lower eukaryote Physarum polycephalum. Structure 11: 75-85.

42. Blanchard H, Grochulski P, Li Y, Arthur JS, Davies PL, et al. (1997) Structure of a calpain Ca(2+)-binding domain reveals a novel EF-hand and Ca(2+)-induced conformational changes. Nat Struct Biol 4: 532-538.

43. Hosfield CM, Elce JS, Davies PL, Jia Z (1999) Crystal structure of calpain reveals the structural basis for Ca(2+)-dependent protease activity and a novel mode of enzyme activation. EMBO J 18: 6880-6889.

44. Moldoveanu T, Gehring K, Green DR (2008) Concerted multi-pronged attack by calpastatin to occlude the catalytic cleft of heterodimeric calpains. Nature 456: 404-408.

45. Hanna RA, Campbell RL, Davies PL (2008) Calcium-bound structure of calpain and its mechanism of inhibition by calpastatin. Nature 456: 409-412.
